# Experimental Optimization of Surface Quality and Process Stability in Robotic Milling of Corian and AlMgSi

**DOI:** 10.3390/ma19214454

**Published:** 2026-10-23

**Authors:** Lukáš Hanko, Peter Križan, Tomáš Tóth, Michal Svátek, Miroslava Švecová, Stanislav Strigáč, Lukáš Pajtinka

**Affiliations:** Institute of Production Engineering and Quality, Faculty of Mechanical Engineering, Slovak University of Technology in Bratislava, 812 31 Bratislava, Slovakia; peter.krizan@stuba.sk (P.K.); tomas.toth@stuba.sk (T.T.);

**Keywords:** robotic milling, industrial robot, surface roughness, vibrations, response surface methodology (RSM), Corian, AlMgSi, modal analysis, machining optimization, full factorial design

## Abstract

**Highlights:**

- Experimental investigation of robotic milling parameters for Corian and AlMgSi.
- RSM identified favorable machining conditions within the investigated parameter ranges.
- Feed per tooth significantly influenced the resulting machined surface roughness.
- Modal analysis supported spindle speed range selection by identifying critical frequency regions.
- Material-specific machining conditions affected surface quality and vibration levels.

**Abstract:**

Robotic milling offers a flexible alternative to conventional CNC machining centers; however, its wider application is limited by the lower structural stiffness of industrial robots and their increased sensitivity to vibrations. The objective of this study was to experimentally investigate the influence of robotic milling process parameters on machined surface quality and vibration intensity during the machining of the Corian polymer composite and AlMgSi aluminum alloy. The experiments were conducted using an ABB IRB 1100 industrial robot equipped with a high-speed milling spindle. Prior to the machining experiments, an experimental modal analysis of the robotic system was performed under static conditions to identify its dominant natural frequencies and potentially critical frequency regions. These results were used as one of the criteria for selecting the initial spindle speed ranges, together with the spindle operating limitations and preliminary machining tests. A full factorial experimental design was subsequently applied separately to each material to investigate the effects of spindle rotational frequency, feed per tooth, and cutting depth. Vibrations were evaluated using RMS values obtained from triaxial accelerometers, while surface quality was assessed by measuring surface roughness using a Keyence optical microscope. Response Surface Methodology was used to model the relationships between the machining parameters and the investigated responses and to identify favorable machining conditions within the investigated parameter domains. The results showed material-dependent differences in process behavior and model performance. In particular, the AlMgSi surface roughness model exhibited limited predictive capability, and its predicted favorable condition should therefore not be interpreted as a reliably established or experimentally validated optimum. The results provide a basis for selecting material-specific machining conditions aimed at reducing vibration levels and improving surface quality in robotic milling.

## 1. Introduction

Robotic milling is an advanced machining technology in which the cutting tool is guided by an industrial robot instead of a conventional machine tool. In recent years, interest in the use of robotic systems in machining has been growing, primarily due to their high flexibility, large working area, and lower acquisition costs compared to conventional CNC machining centers [1]. Robotic milling is primarily used for machining large-sized parts, composite materials, plastics, and wood, as well as for finishing operations in the automotive, aerospace, and mechanical engineering industries [2]. Table 1 below illustrates the various fields of application for robotic milling.

**Figure 1:**
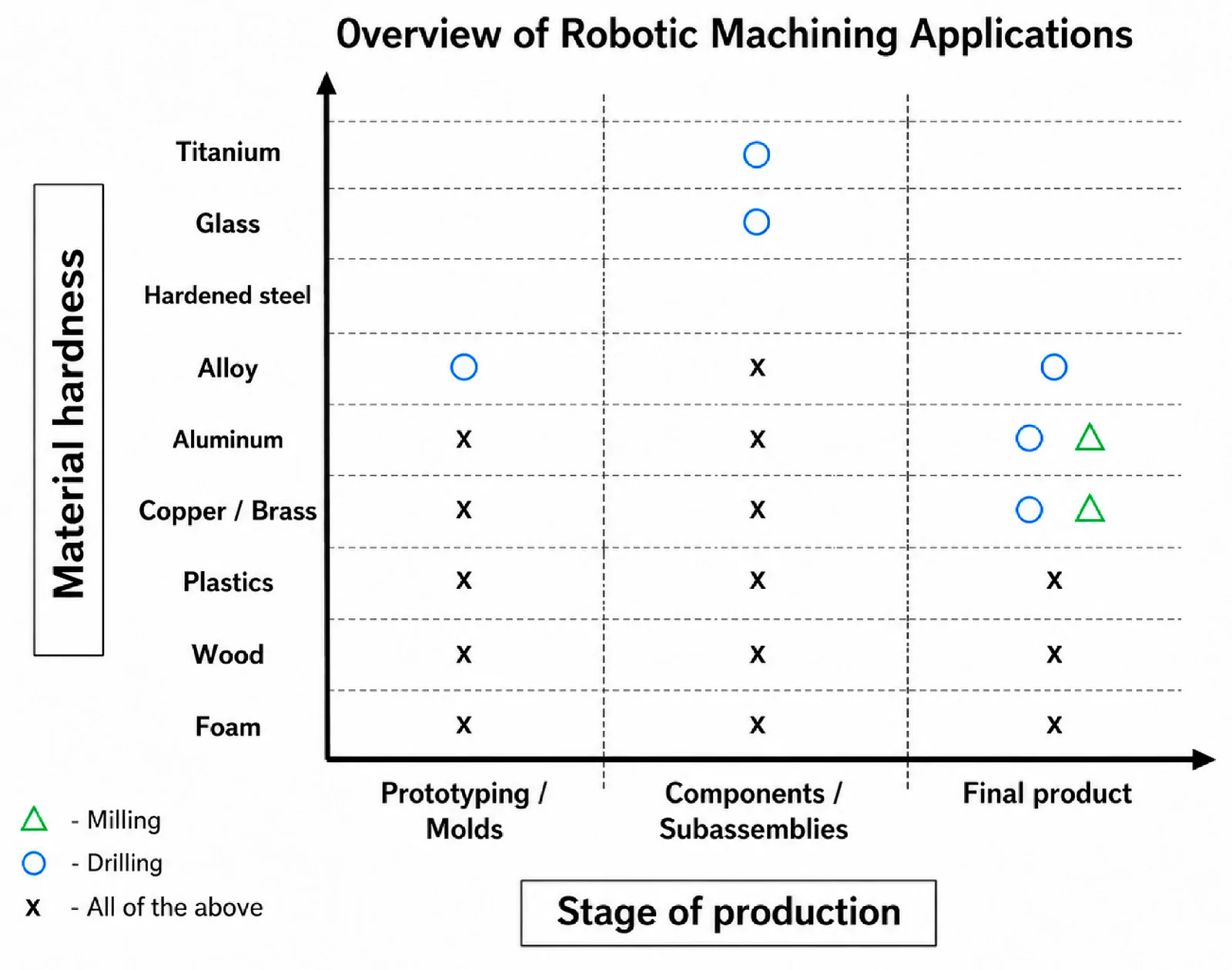
Machining of materials of varying hardness using a robotic arm at specific stages of production [6].

Despite several advantages, the use of industrial robots for machining is associated with a number of technical limitations. Compared to conventional machine tools, robotic manipulators have lower structural rigidity, which results in greater sensitivity to vibrations during the machining process. Furthermore, the dynamic properties of the robotic system vary depending on the configuration of the robotic arm and the position of the workpiece within the workspace. These variations can significantly affect the quality of the machined surface, manufacturing accuracy, and the stability of the milling process. To ensure a high-quality and stable robotic milling process, it is therefore necessary to analyze, not only the technological parameters of machining, but also the kinematic and dynamic properties of the robotic system [3]. In particular, the position of the workpiece within the robot’s working envelope, the configuration of the manipulator’s individual axes, and the natural frequencies of the entire system play a significant role. Determining these properties makes it possible to identify areas of the workspace suitable for performing machining operations and to select process conditions that minimize vibrations and improve the quality of the machined surface. The properties of the material being machined also have a significant impact on the final machining quality. Due to the lower rigidity of the robotic structure, robotic milling is not suitable for machining hard metallic materials at high material removal rates [4]. Therefore, one of the important aspects of robotic milling is the appropriate selection of the workpiece material and machining conditions so that the limitations of the robotic system are taken into account while achieving the required surface quality [5]. Figure 1 below illustrates the possibilities of robotic machining, including individual machining processes and materials suitable for machining using industrial robots.

Conventional CNC machine tools are widely used for multi-axis machining of components in the automotive, mechanical engineering, aerospace, and energy industries. Their high structural stiffness and machining accuracy provide stable conditions for precision machining and enable the production of complex components using various machining processes, including milling, drilling, turning, and grinding. However, conventional CNC machine tools are generally associated with high acquisition and operating costs, and their fixed machine structure and working envelope may limit the machining of very large components or surfaces with difficult accessibility [6]. Industrial robots represent a viable alternative to conventional CNC machine tools, particularly in applications where a large working envelope, high flexibility, and access to geometrically complex surfaces are required [7]. Compared with conventional CNC machine tools, robotic systems generally have lower acquisition costs and provide greater kinematic flexibility. Their working envelope can be extended by integrating additional external axes, and several robots can operate within a shared production environment. These characteristics make robotic systems suitable for flexible manufacturing and the machining of large or geometrically complex components. The increasing use of industrial robots is also associated with their flexibility, functionality, potential for increasing productivity, and ability to perform operations under demanding working conditions while reducing direct operator exposure [8]. However, one of the main limitations of industrial robots in machining applications is their relatively low structural stiffness, which directly affects machining accuracy and process stability. The stiffness of an industrial articulated robot has been reported to be below 1 N/µm, whereas the stiffness of a conventional CNC machine tool can exceed 50 N/µm. This substantial difference makes robotic systems considerably more susceptible to deformation caused by machining forces. Deflections of the robot structure and end-effector during machining can subsequently contribute to increased vibration, deterioration of the machined surface quality, and reduced geometric accuracy. The dynamic behavior of a robotic machining system is also strongly dependent on the configuration of the manipulator. Robot stiffness can vary with manipulator posture, joint configuration, tool position, and workpiece placement within the working envelope. Consequently, machining parameters commonly applied in conventional CNC processes cannot always be transferred directly to robotic milling and should be adapted with respect to the dynamic characteristics of the robotic system [7]. 

This makes the selection of machining parameters particularly important, since inappropriate cutting conditions can increase vibrations and reduce the resulting surface quality. Several approaches have been investigated to reduce the influence of the lower stiffness of industrial robots. These include increasing the structural stiffness of the manipulator, applying advanced control and compensation strategies, and adapting machining conditions to the dynamic characteristics of the robotic system. Increasing the cross-section of structural robot components can improve stiffness; however, this approach also increases the moving mass and can consequently deteriorate the dynamic performance of the manipulator. The application of lightweight materials with high specific stiffness represents another possible approach to improving the structural behavior of robotic manipulators. In addition to structural modifications and compensation methods, the appropriate selection of machining conditions can contribute to improved process stability. Previous research has proposed the use of small-diameter cutting tools combined with high spindle speeds as one approach to reduce cutting forces and compensate for the lower stiffness of articulated robots. High-speed machining has also been investigated in robotic milling of aluminum alloys, where suitable cutting frequencies can help limit the influence of structural resonance and provide more stable machining conditions with reduced chatter and vibration [8]. These findings indicate that the selection of spindle speed, tool geometry, feed, cutting depth, and other technological parameters in robotic milling should consider not only the properties of the workpiece material but also the dynamic characteristics and stiffness limitations of the robotic system. The aim of this study was to experimentally analyze the influence of robotic milling process parameters on the surface roughness of the machined surface and the vibration level of the robotic system during the machining of the Corian polymer composite and the AlMgSi aluminum alloy. A separate experimental plan was created for each material, with the ranges of technological parameters determined individually based on the mechanical and technological properties of the material, the stability of the robotic system, and the performance capabilities of the spindle used. Therefore, the objective was not to directly compare the two materials under identical cutting conditions, but rather to identify the influence of technological parameters and determine favorable machining conditions for each material separately using the Response Surface Methodology (RSM).

## 2. Materials and Methods

Experimental research on robotic milling was conducted using an ABB IRB 1100-4/0.58 industrial robotic arm (ABB AB, Västerås, Sweden). This manipulator belongs to the category of articulated robots and consists of six rotational joints, providing six degrees of freedom. The individual axes are driven by servomotors and arranged in a serial kinematic chain, in which each axis supports the subsequent part of the robot structure. This configuration provides high flexibility and maneuverability and enables the robot to reach different positions and orientations within its workspace. Articulated robots are therefore suitable for a wide range of manufacturing applications and can adapt to different technological operations, workpiece geometries, and production requirements [9]. The ABB IRB 1100-4/0.58 used in this study has a nominal payload capacity of 4 kg, an additional armload capacity of 0.5 kg, a maximum reach of 0.58 m, and a nominal manipulator mass of approximately 21.1 kg. According to the manufacturer, the robot provides a pose repeatability of 0.01 mm and a path repeatability of 0.05 mm under ISO 9283 [10] test conditions. The working ranges of axes A1–A6 are ±230°, −115° to +113°, −205° to +55°, ±230°, −125° to +120°, and ±400°, respectively. The corresponding maximum rotational velocities are 460, 360, 280, 560, 420, and 750°/s. For robotic machining, the mechanical installation of the manipulator is particularly important because the dynamic behavior of the system is influenced by the stiffness of its supporting structure. ABB recommends a minimum foundation resonance frequency of 22 Hz and rigid mounting of the manipulator. The manufacturer also indicates that mechanical disturbances can affect robot and tool accuracy and identifies robot resonance frequencies in the region of approximately 10–20 Hz. Consequently, rigid installation and appropriate selection of the machining location within the robot workspace are important considerations for maintaining process stability. In the experimental setup, the robotic manipulator was rigidly mounted on a workbench. A work plate was installed within the robot workspace, on which a precision machine vise was securely mounted to provide sufficient rigidity and repeatability of workpiece clamping. The machining position within the robot workspace was selected based on preliminary positional tests and was subsequently kept constant during the machining experiments. The workpieces investigated in the experimental phase were the polymer composite material Corian and the aluminum alloy AlMgSi. In experimental robotic cutting methods, Corian is a suitable choice due to its combination of mechanical and technological properties. From a materials perspective, it is a “solid surface” composite material consisting of a combination of mineral and polymer components. Approximately 60–70% of its volume consists of a mineral filler, most commonly aluminum trihydroxide (Al(OH)_3_), derived from bauxite. This component provides the material with hardness, dimensional stability, and good machinability. The remaining approximately 30–40% consists of a polymer matrix based on acrylate resin, specifically polymethyl methacrylate (PMMA), which ensures the material’s cohesion, toughness, and thermoformability [11]. Table 2 lists the mechanical properties of Corian.

The AlMgSi alloy belongs to the group of heat-treatable 6xxx series aluminum alloys, with magnesium and silicon as its main alloying elements. This alloy is characterized by low density, a favorable strength-to-weight ratio, and good corrosion resistance. A key property of the AlMgSi alloy is the ability to enhance its mechanical properties through heat treatment, particularly precipitation hardening. The material also exhibits good formability, machinability, and weldability, which allows for its efficient processing in the manufacture of complex structural components. AlMgSi is based on an aluminum matrix, with magnesium (Mg) and silicon (Si) as the main alloying elements. The magnesium content typically ranges from 0.35 to 1.2 %, and the silicon content ranges from 0.3 to 1.3 %. In addition to these elements, small amounts of manganese (Mn), copper (Cu), iron (Fe), chromium (Cr), zinc (Zn), and titanium (Ti) may also be present, which influence the material’s mechanical properties, corrosion resistance, and machinability. The remainder of the composition consists of aluminum (Al), whose content is typically greater than 95 %. Table 3 lists the mechanical properties of the AW-6060 T6 material [13].

**Figure 2:**
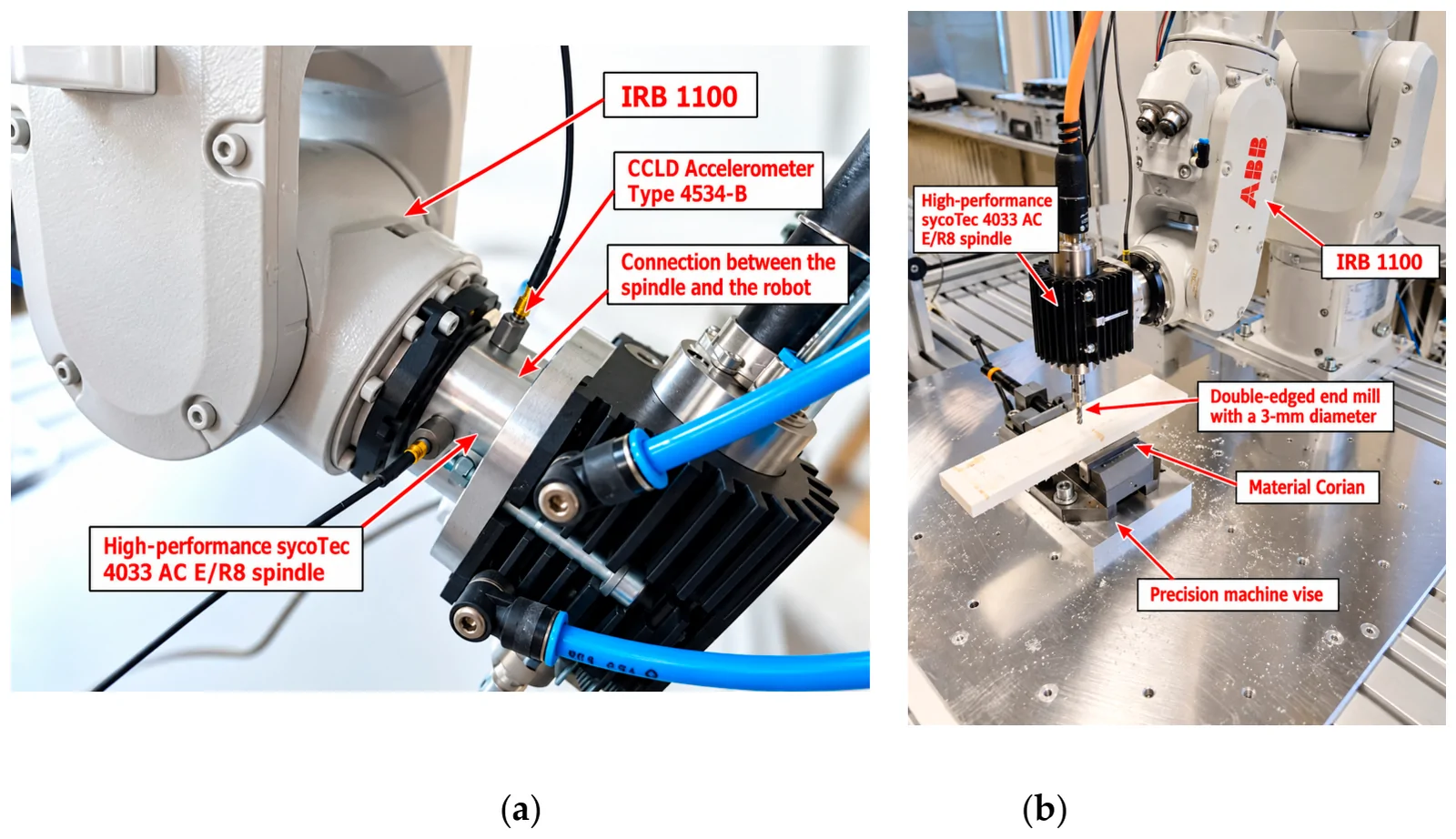
View of a robotic workplace with an ABB IRB 1100 industrial robot with a milling spindle installed. (**a**) View of the spindle flange and the placement of the accelerometers, (**b**) a robotic arm mounted on a workbench along with the material.

The operating speed was controlled by a high-performance SycoTec 4033 AC-ER8 electric spindle (SycoTec GmbH & Co. KG, Leutkirch im Allgäu, Germany) with a power output of 500 W and a speed range of 5000 to 60,000 rpm. Speed control was achieved using an electronic spindle control unit, which allowed for precise adjustment of the desired operating speeds during the experiments. The main rotary cutting motion was provided by a Double-edged VHM micro end mill (Hoffmann Group, Munich, Germany, No. 201140 3X6), secured in the spindle using a collet clamping system. The end mill had a diameter of 3 mm, two cutting edges, a helix angle of 30°, a cutting-edge length of 4.6 mm, a functional length of 6 mm, a shank diameter of 4 mm, and an overall length of 45 mm. The tool was manufactured with sharp cutting edges without a specified corner radius. The cutting portion of the tool was made of monolithic cemented carbide (VHM – Very Hard Material) and coated with a DLC (Diamond-Like Carbon) layer, which reduces friction between the tool and the workpiece and helps extend the tool’s service life. Three CCLD Accelerometer Type 4534-B units were used to measure acceleration values. The Type 4534-B offers a wide frequency range from 0.2 Hz to 12.8 kHz and low noise levels during measurements at low signal levels. It also exhibits low sensitivity to environmental factors, making it suitable for use in changing or demanding conditions. The accelerometers were mounted on a flange designed specifically for this purpose, which was located between the robot’s end-effector and the spindle clamping joint. The flange was designed to allow the accelerometers to be mounted inside it. A total of three accelerometers were mounted on the flange, with each sensor recording acceleration values along the X, Y, and Z axes. The coordinate axes were defined with respect to the machining orientation and remained unchanged throughout all experiments. The X axis corresponded to the tool feed direction, the Y axis represented the transverse direction, and the Z axis was oriented perpendicular to the machined surface. Figure 2 shows the entire working system of the robotic milling cell.

The Corian and AlMgSi workpieces had identical initial dimensions of 100 × 50 × 12 mm (length × width × height). After machining each groove, the workpiece was released from the vise and repositioned so that the subsequent groove was machined on a previously unmachined area. This procedure allowed the same tool trajectory and robot configuration to be maintained for all experimental runs. Consequently, the workpiece length was not a limiting parameter of the individual machining tests but primarily provided sufficient space for successive repositioning and machining of multiple grooves.

### 2.1. Proposal for an Experimental Design

**Figure 3:**
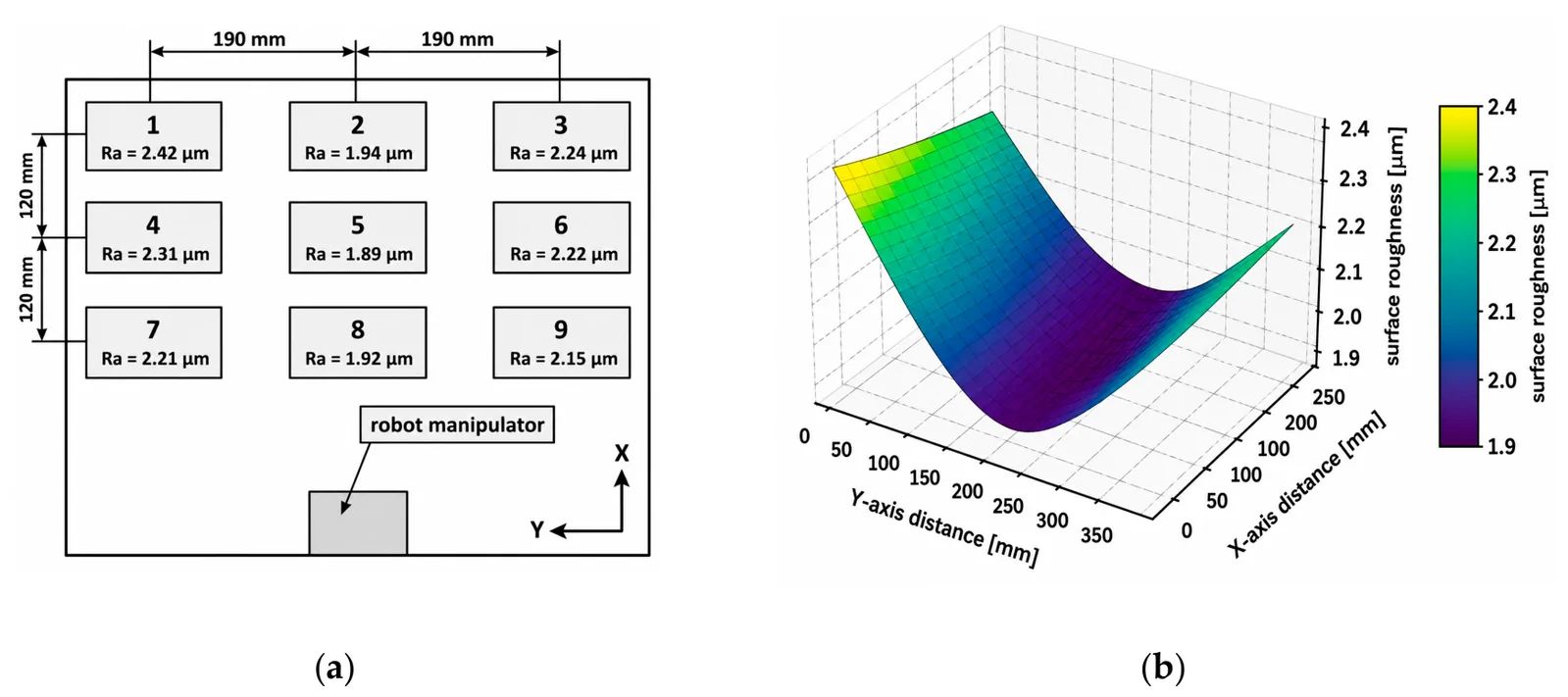
Results of the screening experiment for various positions of the workpiece. (**a**) Roughness values at individual positions, (**b**) a graphical representation of increasing roughness.

The experimental plan for robotic milling consisted of designing the machining parameters, determining the position of the workpiece within the robotic manipulator’s working envelope, and establishing the levels of the individual machining parameters. The proposed positions of the workpiece were based on the results of screening experiments. In this part of the experiment, the cutting tool trajectory was analyzed in nine different positions, which were arranged in a regular grid with equal spacing along the X and Y axes. The Z axis extension of the tool tip was set to the same value for all positions to ensure comparable measurement conditions. Based on the preliminary results of the screening experiments, position number 5 was selected for the main experiment, as it exhibited the most suitable conditions in terms of machining process stability and the dynamic behavior of the robotic system (Figure 3).

**Figure 4:**
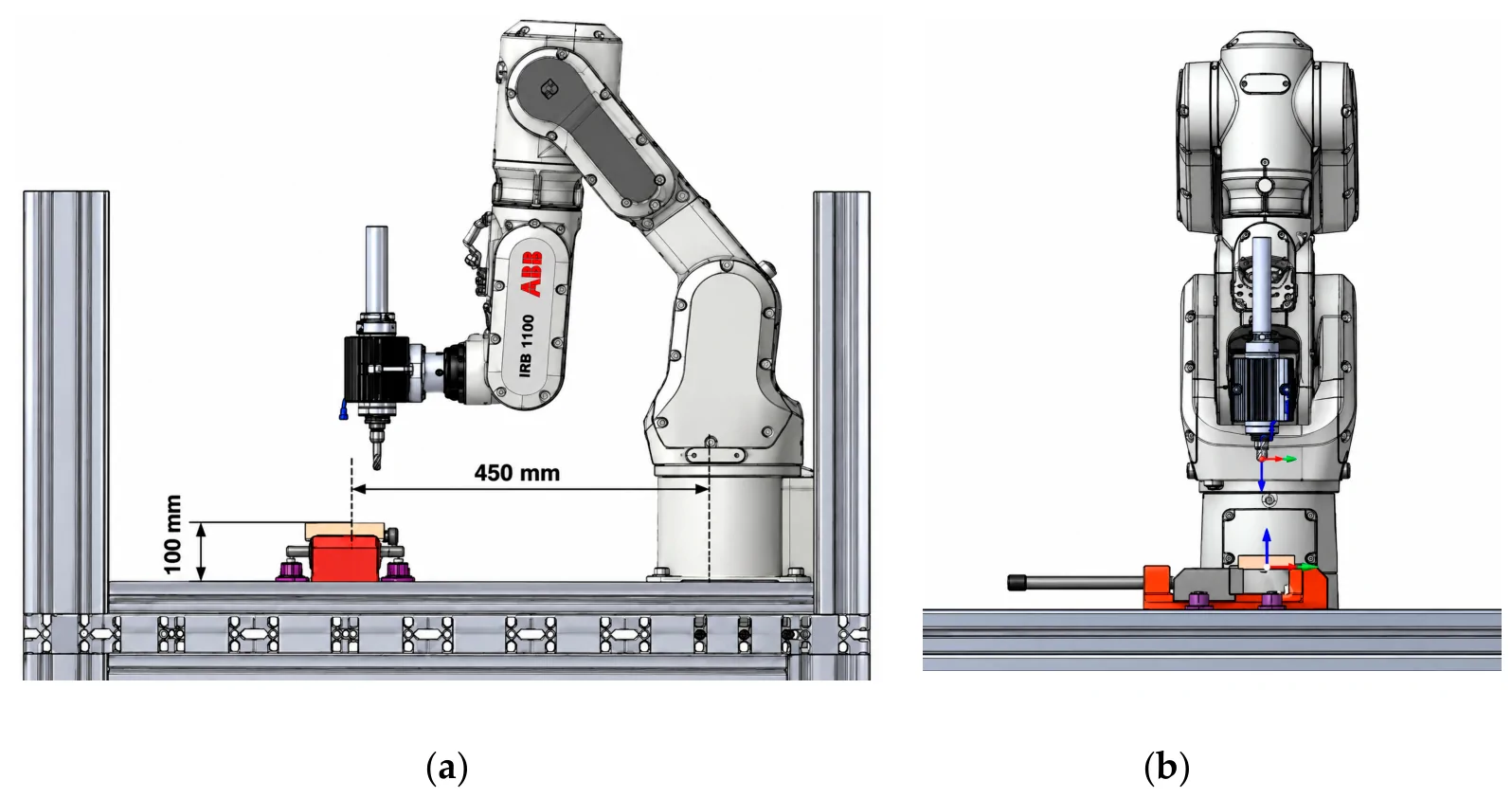
Positioning the workpiece in the robot’s workspace. (**a**) Positioning the workpiece in the XZ plane, (**b**) positioning the workpiece in the XY plane.

The workpiece was clamped using a precision machine vise, which was positioned on the worktable 450 mm from the robot base in the X axis direction. The position of the machine vise was designed so that the cutting tool’s trajectory moved from left to right while intersecting the zero position of the robotic manipulator’s first axis (A1 = 0°). The height of the workpiece in the Z axis was 100 mm relative to the world coordinate system, whose origin was located at the base of the robotic arm (Figure 4).

**Figure 5:**
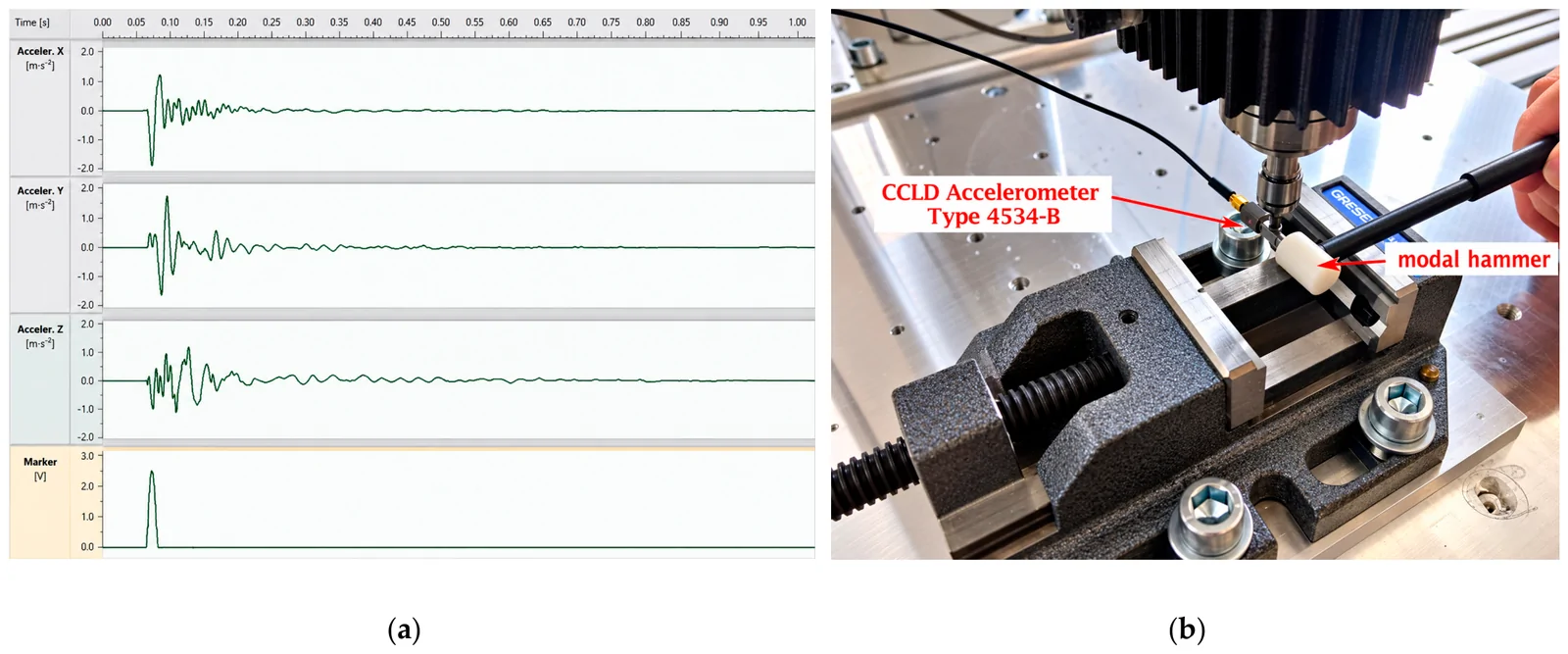
Experimental modal analysis. (**a**) Time-domain spectrum recording generated using a modal hammer, (**b**) excitation of the system using a modal hammer.

The technical literature on robotic milling indicates that machining parameters, particularly the feed per tooth, cutting depth, and spindle speed, significantly influence the resulting quality of the machined surface as well as the dynamic behavior of the robotic structure during the machining process [14,15]. The design of the levels for the individual machining parameters was based on the results of screening experiments, the aim of which was to identify stable operating conditions for conducting the main experiment. As part of the screening experiments, the machining parameters were gradually varied from the minimum to the maximum values recommended by the manufacturers of the tool and the experimental spindle. The first stage of the experimental design involved determining an appropriate spindle operating range. For this purpose, an experimental modal analysis was performed on the entire assembly consisting of the robotic manipulator, milling spindle, flange, and cutting tool. The objective was to identify the natural frequencies and potentially critical frequency regions of the robotic assembly, which were subsequently used as one of the criteria for selecting the initial spindle-speed range. The experimental modal analysis was performed by exciting the robotic structure using a modal hammer (Figure 5). Excitation pulses were applied sequentially in the X, Y, and Z directions to identify the dynamic properties of the system in all principal directions. The system’s response was recorded using an accelerometer mounted on the cutting tool, which made it possible to capture vibrations directly at the point where cutting forces act during machining.

**Figure 6:**
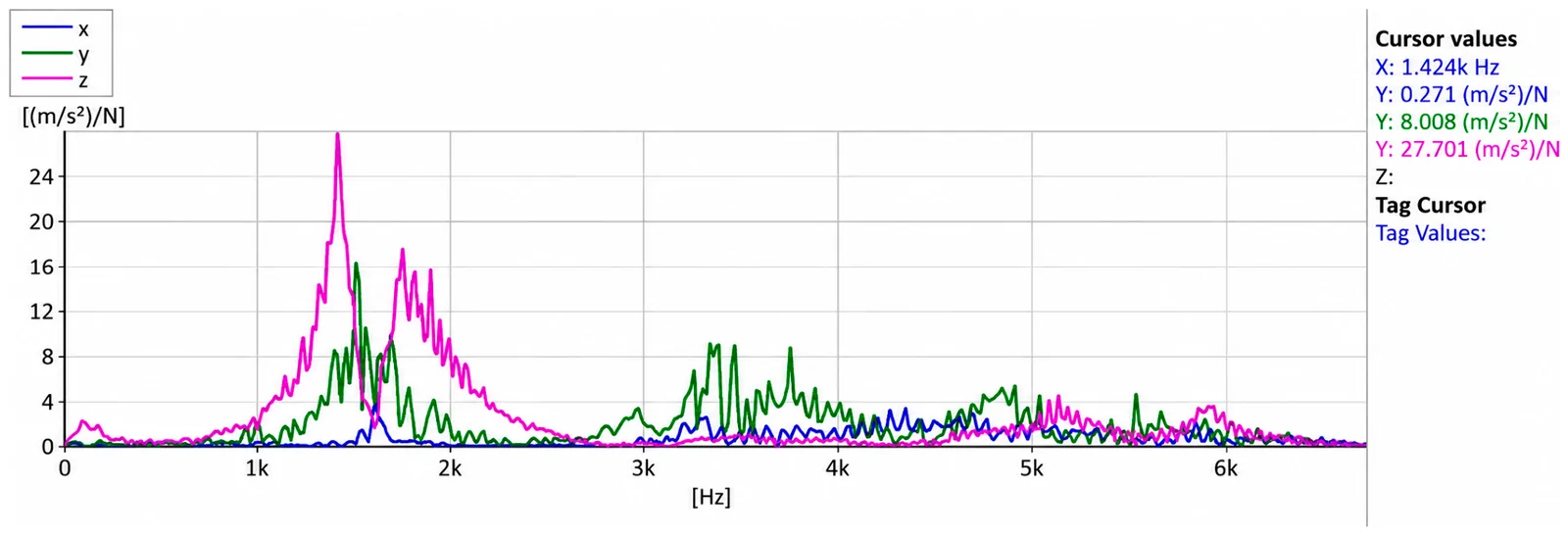
Frequency spectrum of modal analysis.

Figure 6 shows the frequency response (FRF) of the robotic system in the X, Y, and Z directions. A dominant resonance peak was identified in the region of approximately 1.6 kHz, with the highest amplitude of the frequency response recorded in the Z direction. Additional resonance regions are located in the 3–4 kHz and 5–6 kHz bands. The obtained frequency spectrum was subsequently used to support the selection of the experimental spindle-speed ranges with consideration of potentially critical frequency regions.

The operating spindle-speed ranges differed between the two materials due to their different machining characteristics and the operating limitations of the experimental spindle. For Corian, the minimum spindle rotational frequency was set to 566.6 Hz, whereas for the AlMgSi alloy it was increased to 833.3 Hz. During preliminary machining tests of AlMgSi at lower spindle speeds, the increased cutting load caused a pronounced decrease in spindle speed and, under more demanding conditions, could lead to spindle stalling. Therefore, different spindle-speed ranges were defined for the two materials, taking into account the machining behavior of each material, the operating capabilities of the spindle, the dynamic characteristics of the robotic system, and the results of preliminary experiments. The selection of the spindle-speed range was also supported by the experimental modal analysis performed prior to the machining experiments. The modal analysis was conducted under static conditions, without cutting and without tool-workpiece interaction, and was used to identify the dominant dynamic characteristics and potentially critical frequency regions of the robotic system. A dominant natural frequency of approximately 1.6 kHz was identified, together with additional response regions at higher frequencies. When defining the spindle-speed range, these frequency regions were considered in relation to the two-flute geometry of the cutting tool and the corresponding tooth-passing frequency. Thus, the modal analysis served as one of the criteria for selecting the initial spindle-speed range, together with the spindle operating limits and preliminary machining tests. It should be emphasized that the natural frequencies identified by the modal analysis characterize the robotic system under the static conditions of the modal test and are not material-specific frequencies. During actual milling, tool engagement introduces additional interactions associated with the workpiece material, workpiece and clamping stiffness, tool-workpiece contact, and process-dependent cutting stiffness. Therefore, the dynamic response of the coupled system during machining may differ from that measured during the static modal analysis. For this reason, the modal analysis was used to support the selection of the experimental spindle-speed range rather than to determine or predict the actual resonance frequencies during cutting. Based on the selected spindle rotational frequency values, the tool feed rate required for generating the robotic motion instructions was calculated according to machining theory:

Vf = n ∗ z ∗ fz [mm/min](1)

where

n—spindle speed [rpm].
z—number of cutting edges on the tool [-].
fz—feed per tooth [mm].

The feed rate was calculated from the feed per tooth so that the selected feed per tooth was maintained at different spindle speeds. The feed per tooth levels were selected with regard to the cutting-tool geometry, the properties of the machined material, the stability of the milling process, and the results of preliminary screening experiments.

Another parameter for the main experiment was the cutting depth. In this section, the values were gradually varied from the minimum to the maximum. Both parameters were set based on the stability of the robotic structure and the performance characteristics of the experimental spindle. Table 4 and Table 5 below list the levels of the technological parameters for Corian and the AlMgSi alloy.

**Figure 7:**
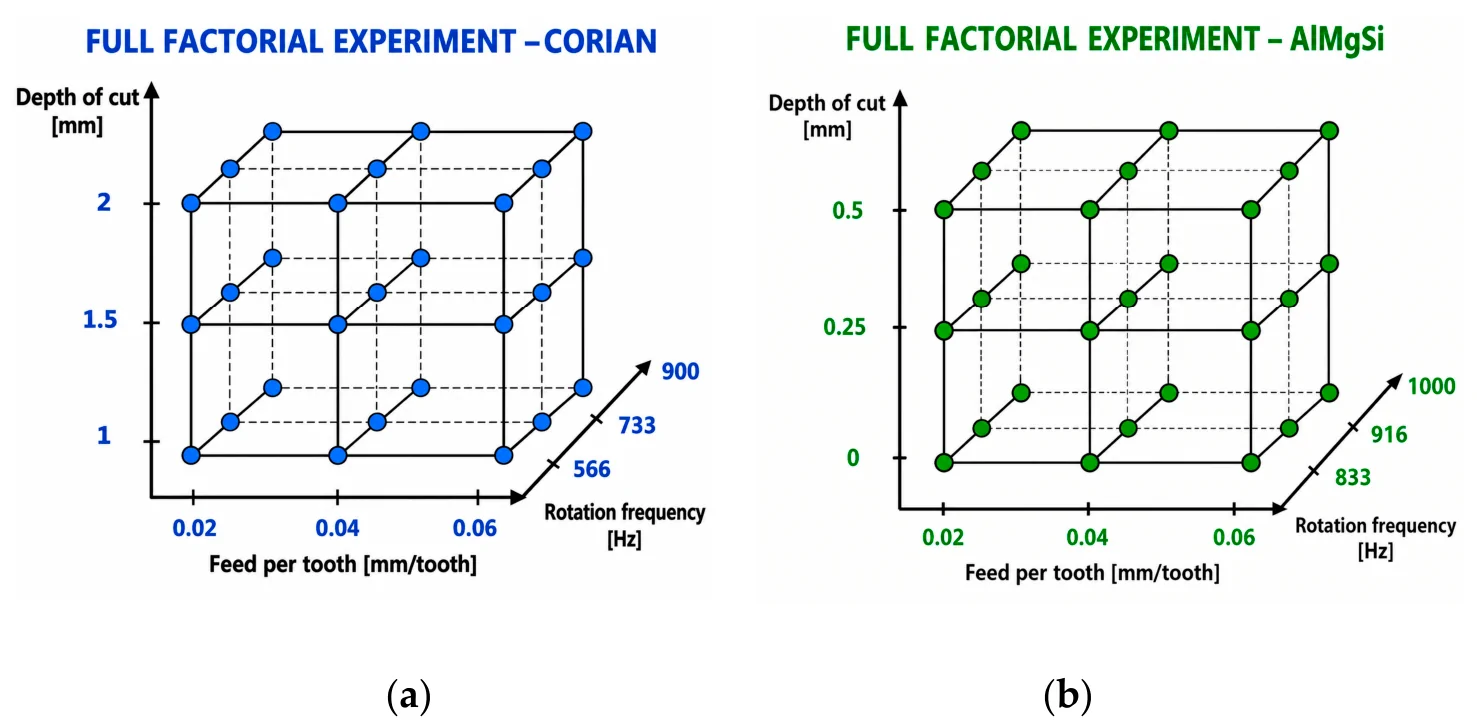
Full factorial design. (**a**) Experimental design for Corian, (**b**) experimental design for AlMgSi.

To effectively analyze the influence of technological parameters on the robotic milling process, a full factorial design methodology was employed in the experimental design (Figure 7). This method represents one of the fundamental approaches to experimental design and enables systematic investigation of the effects of all considered factors as well as their interactions. In a full factorial experiment, all possible combinations of the selected factor levels are tested. This approach provides comprehensive information on the influence of the input parameters on the monitored responses and enables the identification of interaction effects between the process factors. Compared with other experimental design methods, the main advantage of full factorial design is the detailed evaluation of the relationships between the process parameters [16]. The main experimental factors were the machining parameters, namely spindle rotational frequency, feed per tooth, and cutting depth. The parameter ranges were determined separately for each material. The selected levels were based on the results of the experimental modal analysis, the performance limitations of the high-speed spindle, the cutting conditions recommended by the tool manufacturer, and preliminary screening experiments. The purpose of using material-specific parameter ranges was not to compare Corian and AlMgSi under identical cutting conditions, but to investigate each material within a technologically feasible and dynamically stable operating range of the robotic milling system. For Corian, three levels were used for each of the three machining parameters, resulting in a full factorial design with 27 parameter combinations. For AlMgSi, three levels of spindle rotational frequency and three levels of feed per tooth were used, whereas the cutting depth was limited to two experimentally feasible levels, ap = 0.25 and 0.50 mm. During preliminary machining tests of AlMgSi at a higher cutting depth, the increased cutting load caused the spindle to stall. Therefore, higher cutting depth conditions were excluded from the main experimental design because they were outside the feasible operating range of the experimental robotic milling system. Consequently, the AlMgSi experiment consisted of full factorial design with 18 parameter combinations. Furthermore, the same spindle-speed range could not be applied to both materials. During preliminary AlMgSi machining tests at lower spindle speeds, the higher cutting forces caused a pronounced decrease in spindle speed and deterioration of process stability. The experimental designs were therefore defined individually for each material to remain within feasible operating conditions while enabling systematic evaluation and subsequent optimization of the machining parameters.

### 2.2. Robotic Milling

**Figure 8:**
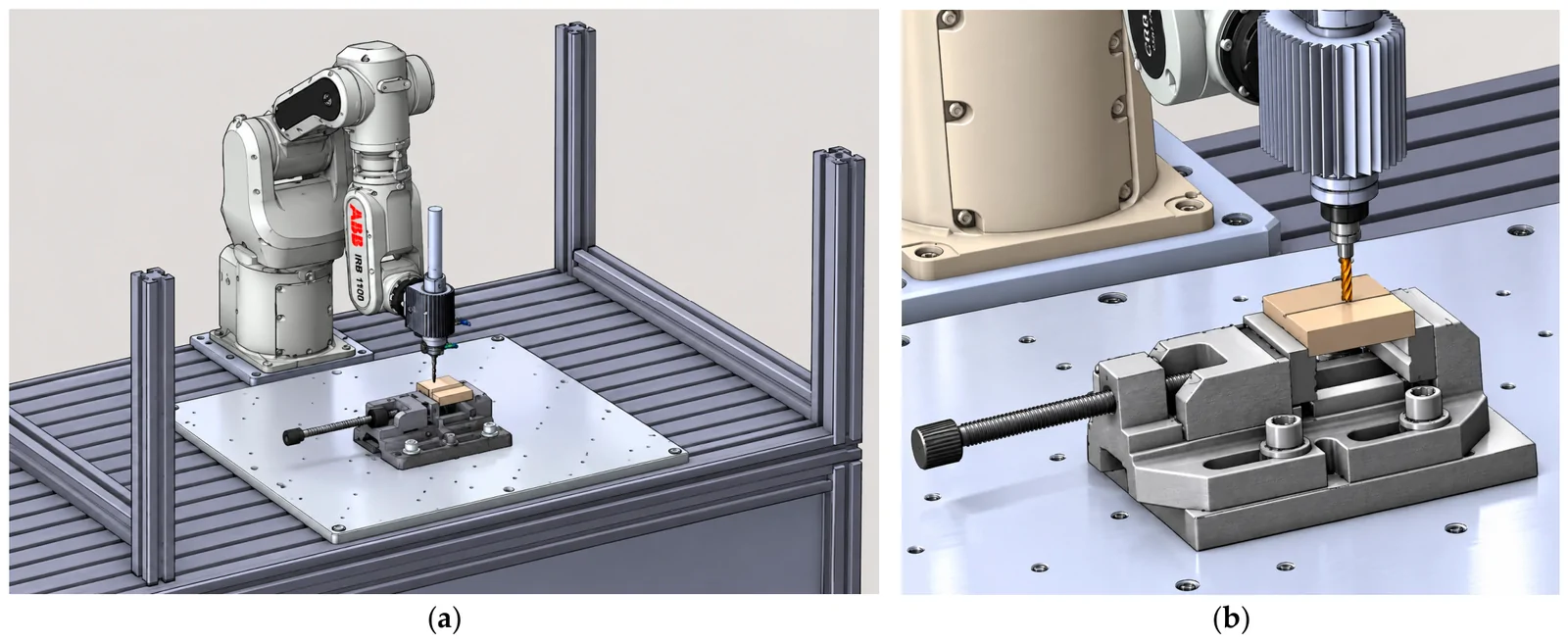
The ENCY program interface. (**a**) A rendered model of a robotic workstation, (**b**) the cutting tool trajectory.

The cutting tool path was created in the ENCY 1 5X simulation environment, version 1.3. The milling process was designed such that, after each groove was milled, the workpiece in the machine vise was moved to a new position, while the tool path remained unchanged. This ensured identical machining conditions for all experiments conducted. The milling process itself was divided into two stages. The first stage consisted of leveling the surface of the workpiece to create a reference plane and define the workpiece’s zero point. After completing this operation, the second stage followed, during which the actual milling of the groove was performed. The tool was first moved along the Z axis to the required cutting depth, and then the machining process was carried out using the specified technological parameters. Figure 8 shows the simulation environment of the ENCY 1 5X v 1.3 program, in which a 1:1 model of the robotic workstation was constructed.

The generated NC code was then imported into the ABB RobotStudio 2026.3 (ABB AB, Västerås, Sweden) simulation environment. In Robot-Studio, auxiliary instructions and control commands were added to the main program. However, these did not affect the cutting tool’s trajectory itself. Their purpose was to ensure improved repeatability. The program was supplemented with time delays during which, after moving from the home position, the tool first pre-positioned itself to the working position in front of the workpiece. This was followed by a dwell time, which allowed the entire mechanical system to stabilize and eliminated transient dynamic phenomena arising from the movement of the robotic manipulator.

### 2.3. Measurement of Dependent Variables

**Figure 9:**
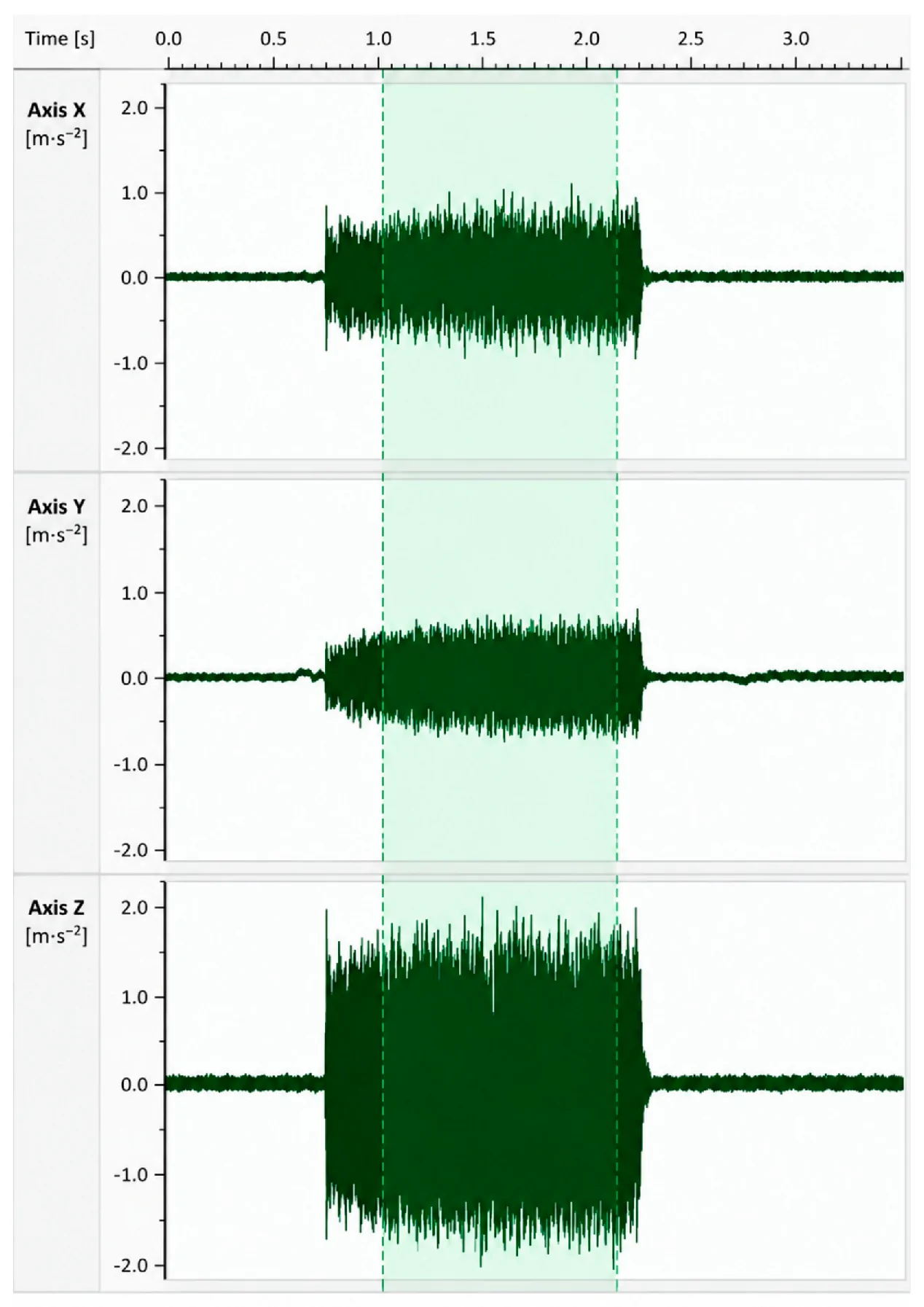
Measurement of the steady-state component of the time-domain vibration record.

The measured dependent parameters in this study were the roughness of the machined surface and the vibrations of the robotic structure. The vibrations were recorded during the milling process using a system of three accelerometers mounted on the flange of the robotic manipulator. The same measurement equipment was also used to perform the experimental modal analysis. During machining, the time history of vibration acceleration was continuously recorded in all three directions of the coordinate system. From the measured time records, the root mean square (RMS) value of the vibrations was then calculated for each direction. Only the steady-state portions of the signal were included in the RMS calculation, while the segments corresponding to the cutting tool’s entry into and exit from the material were excluded from the analysis (Figure 9). This eliminated the influence of transient phenomena and ensured an objective comparison of the individual experiments.

Based on the RMS values obtained for each axis, the total RMS vibration vector V was then calculated, representing the resulting vibration intensity of the robotic structure during the milling process. This quantity was used as the main parameter for evaluating the dynamic behavior of the robotic system during the milling process. RMS represents the effective value of a vibration signal and is one of the basic parameters used to evaluate vibration intensity [12]. It is calculated as the square root of the mean of the squares of the instantaneous values of the signal amplitude over a defined time interval.

(2) $$\mathrm{RMS}=\sqrt{\frac{1}{T}}\int_{0}^{T}{v(t)}^{2}\mathrm{dt}$$

**Figure 10:**
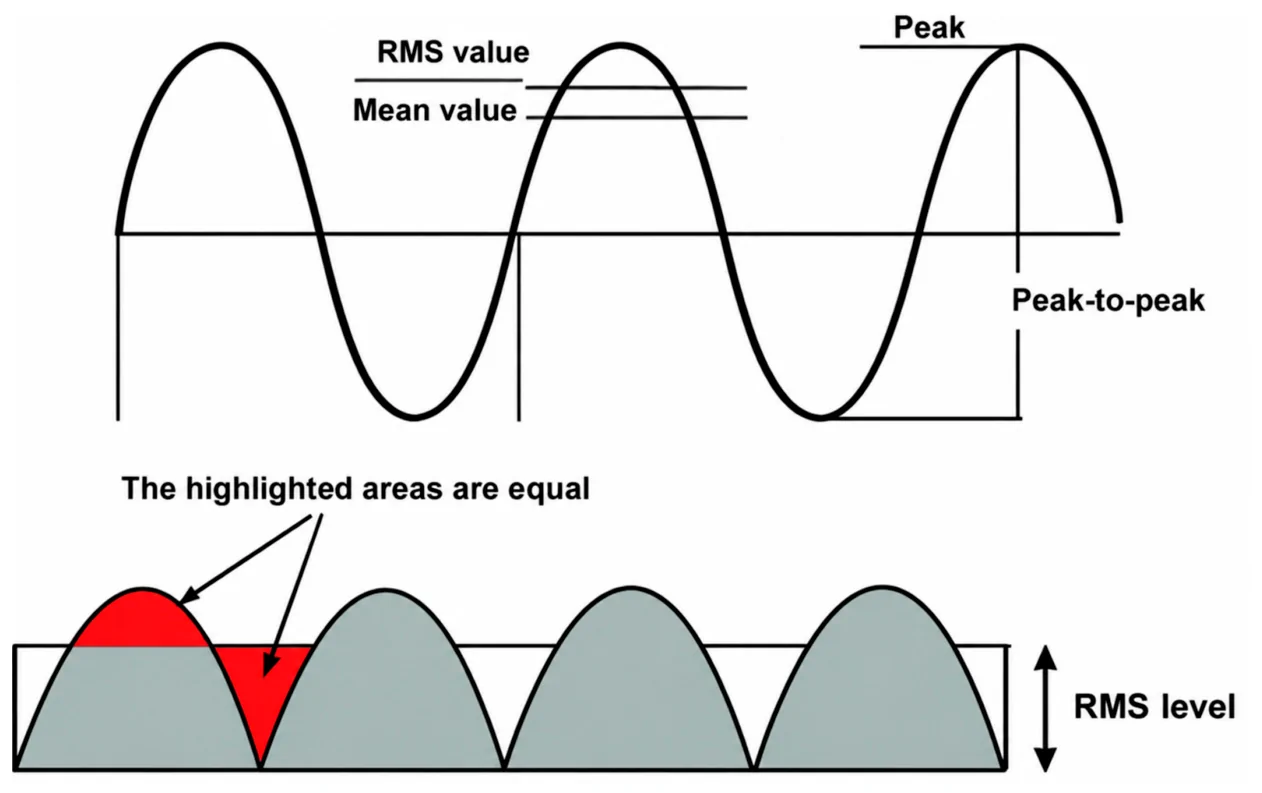
Calculation of the RMS value of a sine wave signal.

For a sinusoidal waveform (Figure 10), the RMS value corresponds to approximately 0.707 times the peak amplitude. However, this relationship applies only to an ideal sinusoidal signal. The advantage of the RMS value is that it takes into account the total energy of the vibration signal regardless of its waveform, and therefore provides an objective indicator of vibration intensity [17]. In this work, the RMS value was used as the main parameter for evaluating the vibrations of the robotic structure during the milling process. The resulting RMS value for all components was calculated using the formula 2.

**Figure 11:**
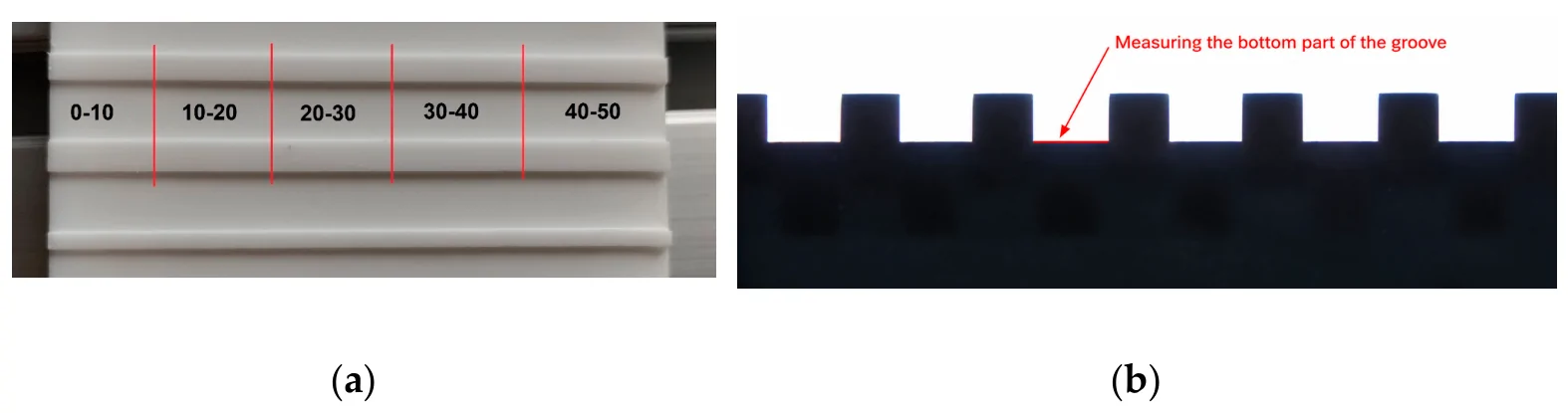
Roughness measurement on samples. (**a**) Identification of a stable area of the indentation, (**b**) measurement of the bottom of the indentation.

Surface roughness was measured at the bottom of each milled groove. As with the vibration measurements, only stable machining areas (area 10–20 and 30–40) were analyzed during the roughness evaluation to ensure the accuracy and comparability of the results obtained (Figure 11).

**Figure 12:**
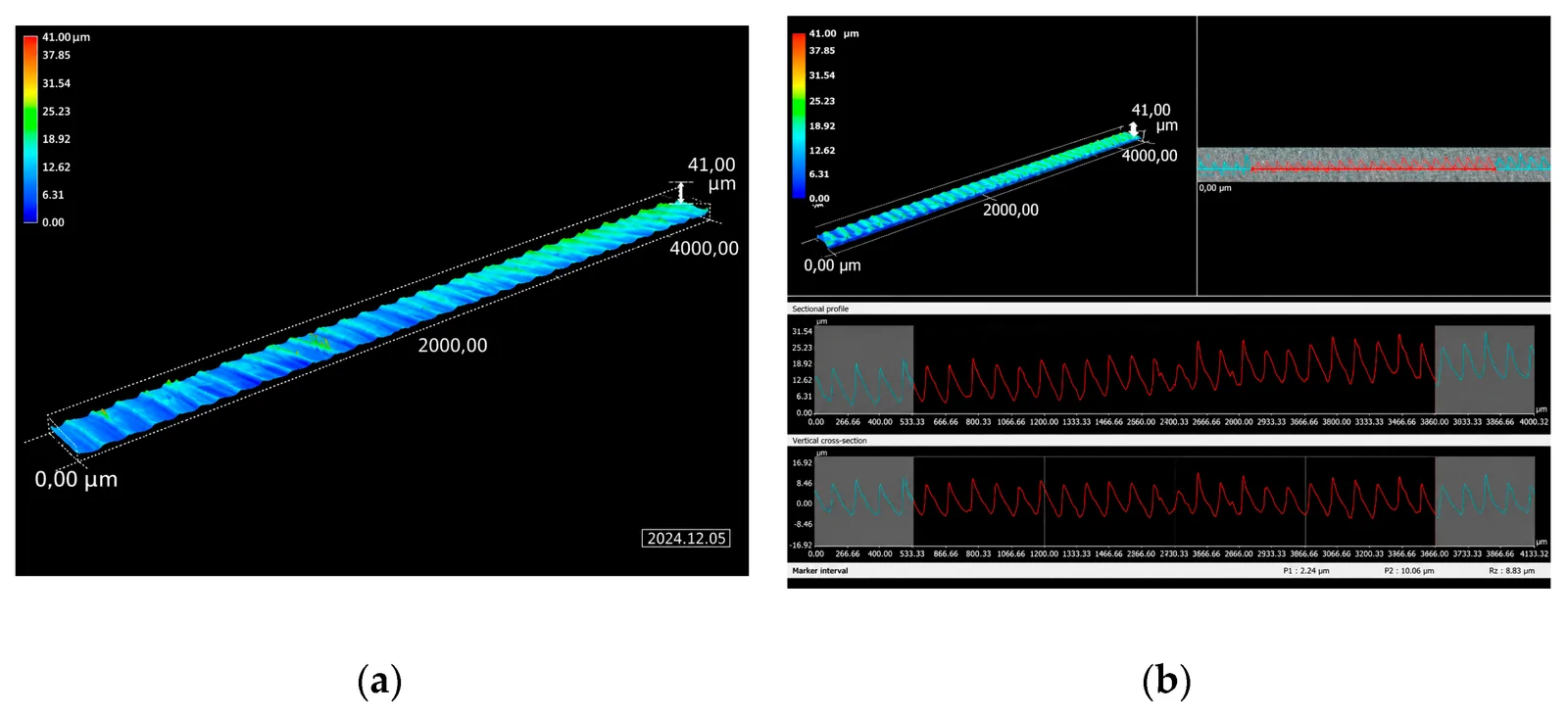
Measurement of linear roughness. (**a**) 3D model of the groove surface, (**b**) measured roughness on 3D models.

A KEYENCE VHX-X1 optical microscope (KEYENCE Corporation, Osaka, Japan) was used to measure surface roughness. This instrument enables non-contact scanning and three-dimensional surface reconstruction. Scanning the bottom of the groove resulted in a precise 3D model of the machined surface. Prior to the actual evaluation, a surface tilt correction was applied to the obtained model to eliminate the influence of inaccuracies in sample positioning during measurement. The roughness evaluation was performed using the linear profile roughness measurement method. The microscope was set to 500× magnification, and the measurement was taken in a stable, milled area of the groove. All measurements were conducted in accordance with ISO 3274:1998 [18], which defines the conditions for measuring profile roughness, including the measured length and the settings for the roughness and waviness filters. The measured profile length was set to 4 mm. The short-wavelength filter λs was set to 2.5 μm, and the filter cutoff wavelength λc was set to 0.8 mm. These settings ensured uniform measurement conditions and allowed for an objective comparison of machined surface quality across the various experimental conditions. Figure 12 shows the surface roughness measurement results obtained using a microscope.

Generative artificial intelligence (ChatGPT-5.6, OpenAI) was used exclusively for visual editing and enhancement of selected figures in the manuscript. The tool was not used for experimental design, data collection, data analysis, or interpretation of the experimental results. The authors reviewed the edited figures and verified their accuracy and consistency with the original scientific data. The authors take full responsibility for the content of the manuscript.

## 3. Results

In this study, the analysis of measured surface roughness and vibration data focused on evaluating the influence of technological parameters and identifying favorable cutting conditions for achieving low surface roughness while maintaining low vibration levels. An RSM model was used for the analysis. This model represents a mathematical-statistical approach designed to model the relationship between process parameters and measured dependent parameters. The analysis of the processed values for the RSM models was performed separately for each material, as the Corian and the AlMgSi alloy were not milled under the same technological settings. A common model incorporating a material factor would therefore not have been methodologically appropriate. The different settings for the technological parameters were based on the dynamic properties of the robotic structure and the material properties. When determining the levels of machining parameters, it was established that they would be set to the maximum values permitted by the robot’s kinematic structure. This means that for each material, the machining parameter levels were selected to be the maximum values allowed by both the properties of the material being machined and the dynamic capabilities of the robotic system. The RSM models were created from the average values of the individual combinations of technological parameters. Corian included 27 combinations with 7 repetitions, and AlMgSi included 18 combinations with 7 repetitions. Table 6 presents the basic characteristics of the RSM models for surface roughness Ra and RMS vibration V for the machined materials Corian and AlMgSi. The quality of the models was evaluated based on the coefficient of determination (R^2^), the adjusted coefficient of determination (Adjusted R^2^), and the root mean square error (RMSE).

For Corian material, the model for predicting surface roughness (Ra) achieved the highest accuracy with an R^2^ value of 0.883 and an Adjusted R^2^ of 0.821, which means that the model can explain approximately 88.3% of the variability in the measured roughness values. The model developed for vibrations (RMS) also achieved good accuracy (R^2^ = 0.788). However, the lower value of the adjusted coefficient of determination (Adjusted R^2^ = 0.675) indicates a slight reduction in predictive ability when the number of model parameters is taken into account. For the AlMgSi material, the most accurate model was the one developed for vibration prediction, which achieved an R^2^ of 0.900 and an Adjusted R^2^ of 0.811. This result demonstrates the model’s very good ability to describe the relationship between machining parameters and vibration intensity. In contrast, the model developed for predicting surface roughness exhibited significantly lower accuracy (R^2^ = 0.546, Adjusted R^2^ = 0.142). Such a low value of the adjusted coefficient of determination suggests that the observed machining parameters alone cannot sufficiently explain the formation of surface roughness during the machining of the AlMgSi alloy. The final quality of the machined surface can also be significantly influenced by other factors, such as the dynamics of the cutting process, cutting tool wear, the properties of the workpiece material, the chip formation process, the rigidity and stability of the workpiece clamping, as well as the rigidity of the robotic system’s mounting to the worktable. Therefore, the AlMgSi Ra model has limited predictive capability and should be interpreted with caution. Its subsequent use in RSM optimization is intended to indicate favorable trends within the investigated parameter domain rather than to provide a robust prediction of the global optimum. Table 7 lists the values measured after the experiment was conducted, showing the average values for vibration and roughness.

For Corian, the lowest experimentally observed roughness was recorded at a rotational frequency of 566.7 Hz, a feed per tooth of 0.02 mm/tooth, and a cutting depth of 1.5 mm, where the mean Ra value was 0.402 µm and RMS V was 13.34 m/s^2^. This combination also provided the lowest combined experimental Score. For AlMgSi, the lowest experimentally observed mean surface roughness of 0.801 µm was obtained at a rotational frequency of 1000 Hz, a feed per tooth of 0.02 mm/tooth, and a cutting depth of 0.25 mm. The experimentally observed combinations in Table 7 are distinguished from the model-predicted solutions presented subsequently in Table 8. Table 8 summarizes the model-predicted machining conditions obtained by RSM for AlMgSi and Corian according to three optimization criteria: minimum surface roughness (Ra), minimum vibration intensity (RMS V), and the combined Ra + RMS V criterion. The table also includes 95% confidence intervals to indicate the uncertainty associated with the model predictions. These results represent model-predicted favorable conditions within the investigated parameter domains and should be distinguished from the experimentally observed combinations presented in Table 7. For the AlMgSi alloy, the RSM model predicted a minimum Ra of 0.857 µm at a rotational frequency of 833.3 Hz, a feed per tooth of 0.02 mm/tooth, and a cutting depth of 0.25 mm. However, due to the limited predictive capability of the AlMgSi Ra model (R^2^ = 0.546, adjusted R^2^ = 0.142), this result should be interpreted with caution as a model-predicted favorable condition within the investigated parameter domain rather than as a reliably established optimum. The lowest experimentally observed mean Ra was 0.801 µm at 1000 Hz, 0.02 mm/tooth, and 0.25 mm (Table 7). For RMS V, the AlMgSi model showed substantially better predictive capability (R^2^ = 0.900, adjusted R^2^ = 0.811) and predicted a minimum of 39.12 m/s^2^ at 1000 Hz, 0.032 mm/tooth, and a cutting depth of 0.50 mm. The predicted conditions for minimum Ra and minimum RMS V in AlMgSi do not coincide, indicating a trade-off between surface quality and process stability. If surface quality is the primary objective, lower feed per tooth and the parameter region associated with lower predicted Ra are preferable. In contrast, if minimizing vibration is the main objective, higher rotational frequency and a feed per tooth of approximately 0.032 mm/tooth are more favorable. When both responses are considered simultaneously, the combined Ra + RMS V criterion provides a compromise between the two objectives. However, because of the limited predictive capability of the AlMgSi Ra model, the combined solution should also be interpreted cautiously within the investigated parameter domain. For Corian, the model predicted a minimum Ra of 0.388 µm at 724.1 Hz, 0.02 mm/tooth, and 1.66 mm cutting depth. The predicted minimum RMS V was 9.114 m/s^2^ at 659.3 Hz, 0.034 mm/tooth, and 1 mm cutting depth. The combined model-predicted solution was obtained at 705.6 Hz, 0.02 mm/tooth, and 1.333 mm cutting depth, with predicted values of Ra = 0.403 µm and RMS V = 21.39 m/s^2^. For Corian, the model predictions were generally close to the experimentally observed favorable conditions, indicating good agreement between the regression model and the experimental data. In contrast, more pronounced differences were observed between the experimental and model-predicted results for AlMgSi surface roughness. This behavior is consistent with the lower predictive capability of the AlMgSi Ra model and the greater variability of the experimental roughness data. Therefore, the predicted AlMgSi Ra minimum should not be considered a validated optimum.

**Figure 13:**
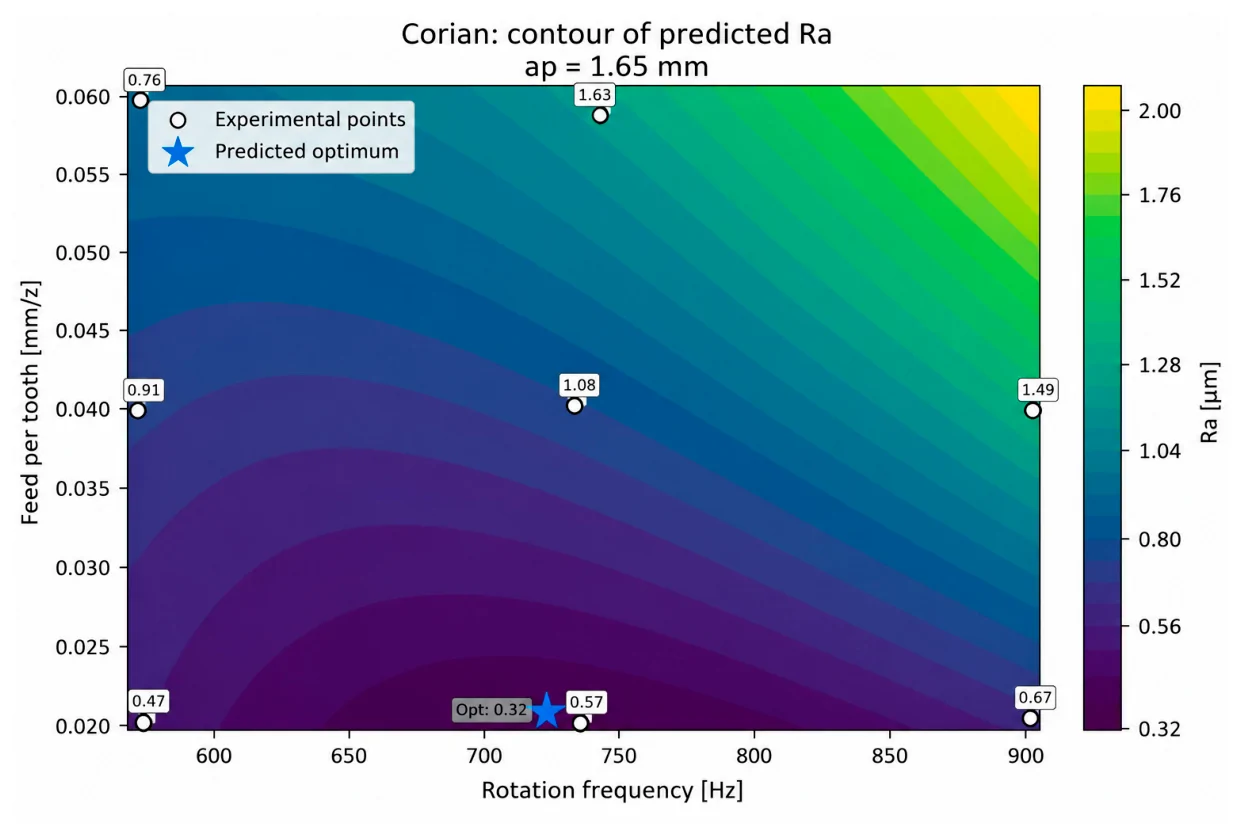
A contour map predicting the surface roughness of Corian at cutting depth of 1.65 mm.

Based on the developed RSM models, the influence of the individual machining parameters on the predicted responses was further evaluated using contour maps. These maps illustrate the predicted surface roughness and RMS V within the investigated parameter ranges for Corian and AlMgSi. The color scale represents the magnitude of the predicted responses, with darker areas indicating lower values and lighter areas indicating higher values. From an initial analysis of the contour map of the predicted surface roughness Ra for the Corian material, it is possible to determine the distribution of the predicted roughness values at a constant cutting depth = 1.65 mm (Figure 13). From the contour lines as well as the results, it is evident that the Ra roughness value is primarily influenced by the technological parameter feed per tooth. Increasing this value leads to a gradual increase in the observed Ra parameter across the entire range of examined rotational frequencies. The influence of rotational frequency is less pronounced and manifests itself primarily at higher feed-per-tooth values. The predicted minimum lies in the region of the lowest feed per tooth and at a rotational frequency of approximately 724 Hz, which corresponds to the optimization results shown in the table, where the minimum predicted Ra roughness of 0.388 μm was achieved.

**Figure 14:**
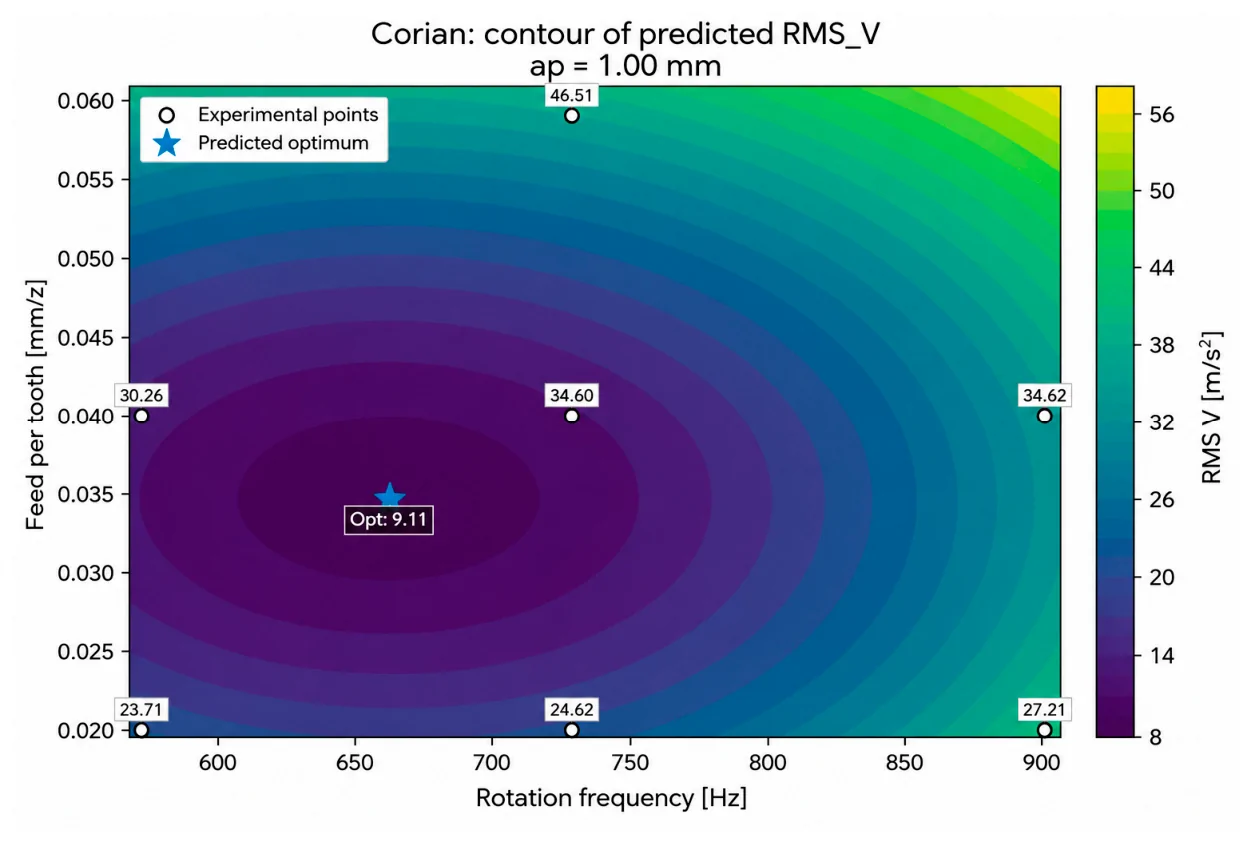
A contour map showing the RMS V value for Corian at cutting depth of 1 mm.

In contrast to surface roughness, the distribution of predicted vibration values exhibits a more pronounced nonlinear character, as can be seen in Figure 14, which shows a contour plot of the predicted RMS V for Corian material. The lowest RMS values are found in the central region of the contour map, where the average rotational frequency combines with a feed per tooth of 0.034 mm/tooth. As the distance from this region increases, the monitored vibration parameter RMS V gradually rises. The optimization identified a minimum vibration level at a frequency of 659 Hz, where the model predicts an RMS V value of 9.11 m/s^2^.

**Figure 15:**
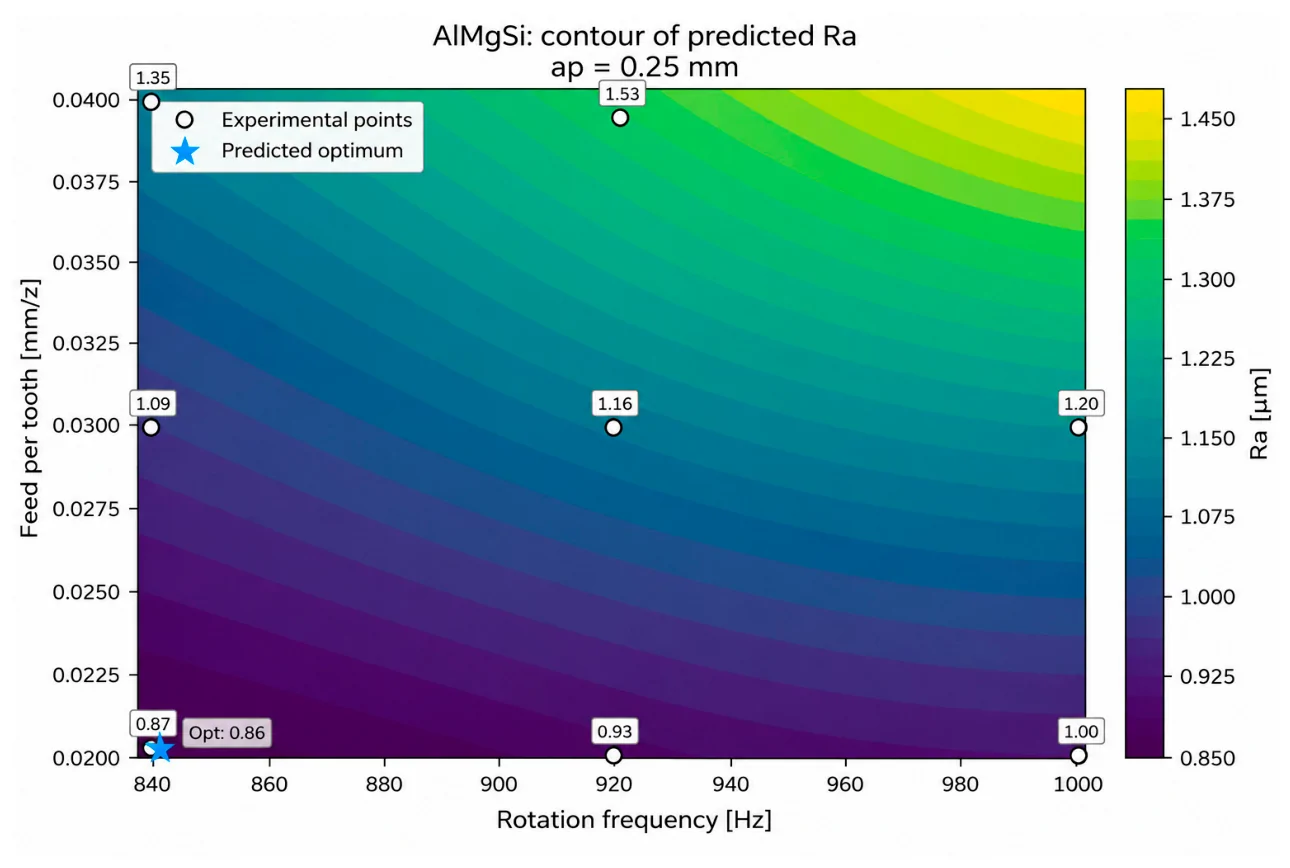
Contour map predicting roughness values for AlMgSi material at cutting depth of 0.25 mm.

The contour plot of the AlMgSi Ra model (Figure 15) indicates a general tendency toward lower predicted roughness at lower feed per tooth. Within the investigated parameter domain, the model predicts its minimum Ra of 0.857 µm at approximately 833 Hz and 0.02 mm/tooth. However, considering the limited predictive capability of the AlMgSi Ra model (R^2^ = 0.546, adjusted R^2^ = 0.142), the contour map should be interpreted primarily as an indication of the modeled response trend rather than as a reliable representation of the exact optimum. The experimental results should therefore remain the primary basis for interpreting surface roughness behavior in AlMgSi.

**Figure 16:**
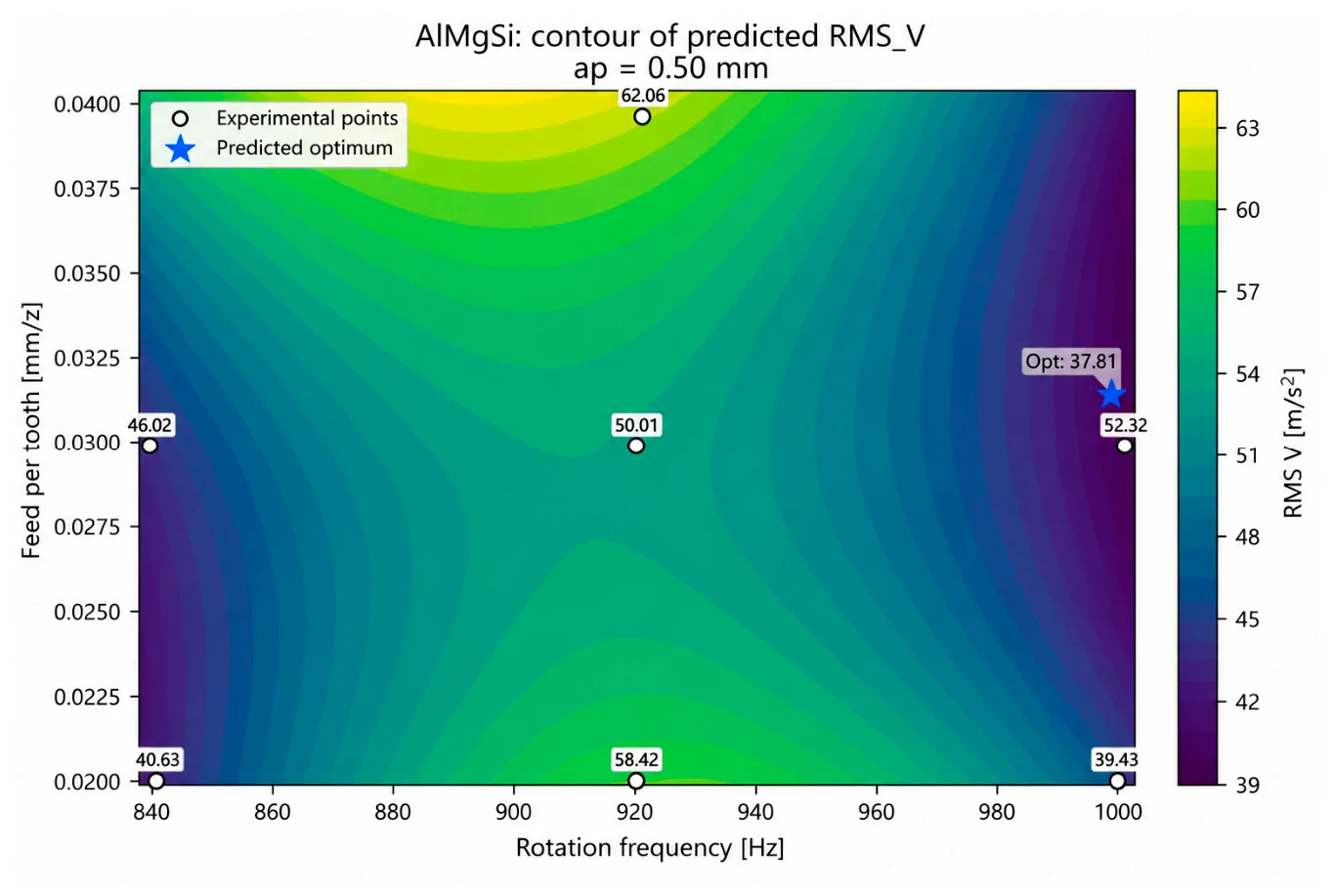
Contour map predicting RMS V values for AlMgSi material at cutting depth of 0.5 mm.

Figure 16 shows the distribution of predicted vibrations during the machining of the AlMgSi alloy. The lowest predicted vibration values occur at a higher rotational frequency of approximately 1000 Hz and a feed per tooth of about 0.032 mm/tooth. This trend suggests that the dynamic response during AlMgSi machining is sensitive to changes in cutting conditions, particularly to the spindle rotational frequency.

**Figure 17:**
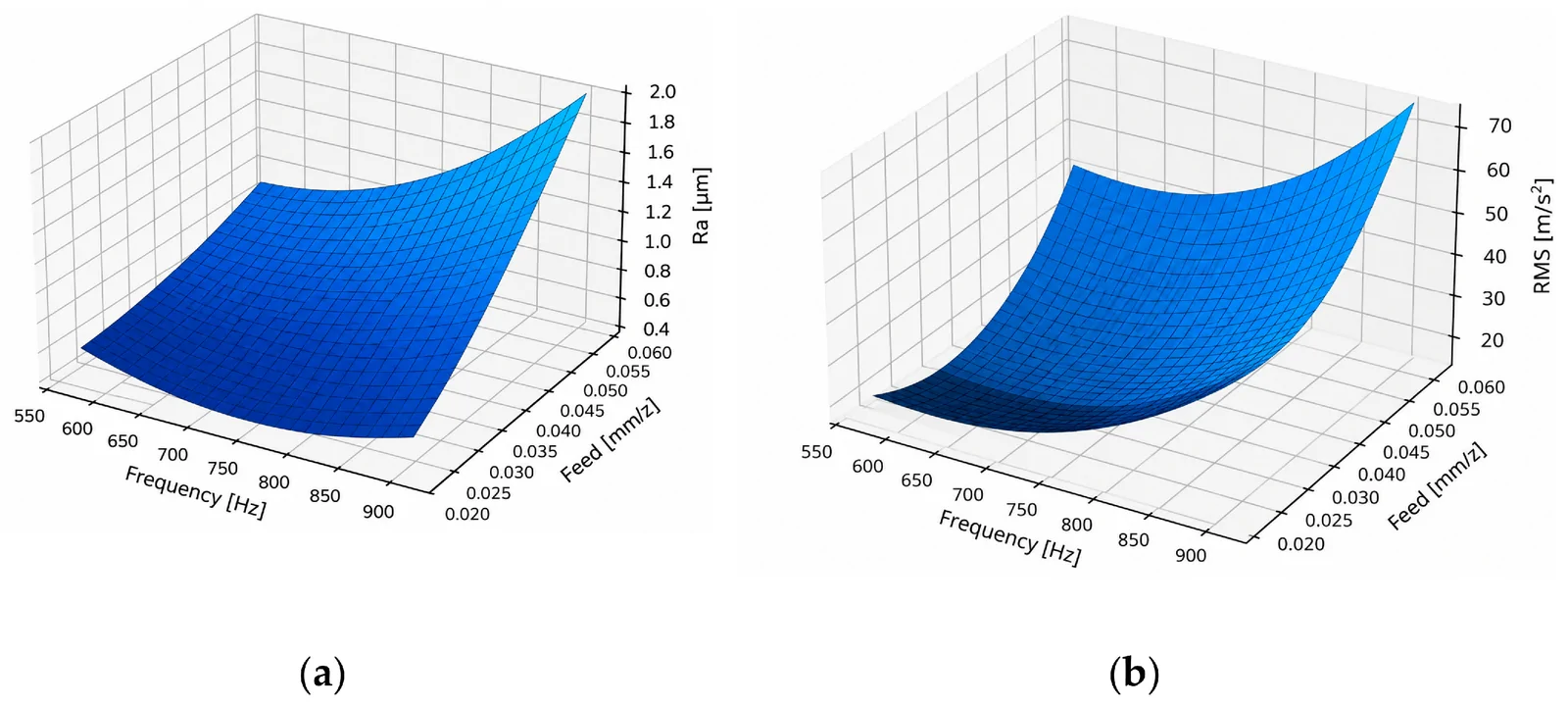
Surface profile for Corian material. (**a**) Roughness (Ra) cutting depth 1.65 mm; (**b**) RMS V and cutting depth 1 mm.

**Figure 18:**
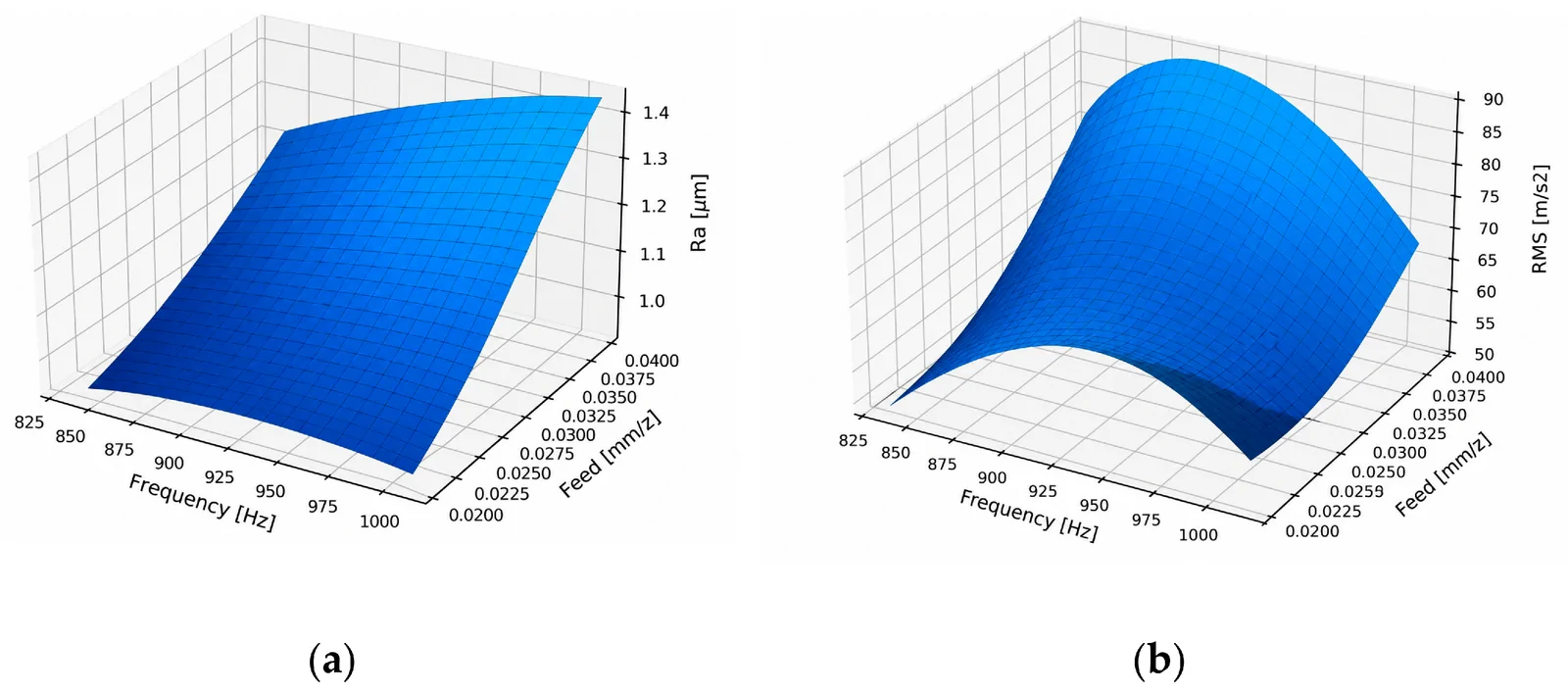
Surface profile for AlMgSi material. (**a**) Surface roughness (Ra) and cutting depth of 0.25 mm; (**b**) RMS V and cutting depth of 0.5 mm.

The optimization process was carried out separately for each material being machined, since, given the performance capabilities of the experimental milling spindle and the rigidity of the robotic system, it was not possible to use the same machining parameters for both materials. The differing mechanical properties of the materials, particularly density, modulus of elasticity, hardness, and strength, significantly influence the course of the cutting process, the magnitude of cutting forces, and the dynamic behavior of the robotic system during milling. For this reason, Corian and AlMgSi were analyzed separately, and a separate predictive model describing the robotic milling process was created for each material. The results of the optimization of the technological parameters are then presented using three-dimensional response surfaces for each material separately in Figure 17 and Figure 18.

## 4. Discussion

### 4.1. Influence of Machining Parameters and Material

The results showed that the response of the robotic milling system depends on both the machining parameters and the properties of the machined material. Corian and AlMgSi exhibited different machining behavior, therefore, separate experimental ranges and regression models were used for each material. The aim was not to directly compare the materials under identical machining conditions, but rather to investigate each material within the technologically feasible operating range of the experimental system. For AlMgSi, a higher spindle speed range and a limited cutting depth had to be used because preliminary experiments showed that higher cutting loads resulted in a significant decrease in spindle speed and, under more demanding conditions, could even cause the spindle to stall. Differences between the materials were also reflected in the quality of the regression models. For Corian, the surface roughness model achieved R^2^ = 0.883 and Adjusted R^2^ = 0.821, while the vibration model achieved R^2^ = 0.788 and Adjusted R^2^ = 0.675. For AlMgSi, the vibration model showed very good agreement with the experimental data (R^2^ = 0.900; Adjusted R^2^ = 0.811), whereas the surface roughness model had considerably lower predictive capability (R^2^ = 0.546; Adjusted R^2^ = 0.142). The low Adjusted R^2^ indicates that the investigated machining parameters explain only a limited proportion of the variability in the surface roughness of AlMgSi. Therefore, the model-predicted parameter combination for minimum Ra cannot be considered a reliably determined or experimentally validated optimum, but only a model-predicted favorable condition within the investigated experimental domain. The lowest experimentally observed Ra value and the model-predicted minimum must therefore be interpreted separately. Higher cutting loads and the compliance of the robotic system may be additional influencing factors; however, their individual effects were not quantified in this study. These results are consistent with the findings of Ni et al. [19], who demonstrated a significant influence of machining parameters on surface quality and the dynamic stability of the system during robotic milling of 7075-T651 alloy. Their results particularly highlighted the influence of spindle speed on surface roughness and the influence of cutting depth on vibration. Similarly, Hanko et al. [20] identified feed and spindle speed as significant factors affecting surface quality. Yan et al. [21] also pointed out that suitable robotic machining conditions depend on the properties of the machined material and cannot be universally applied to different materials. For AlMgSi, the model-predicted conditions for minimum Ra and minimum RMS V did not coincide. The favorable model-predicted condition for Ra was found at a spindle rotational frequency of 833.3 Hz, a feed per tooth of 0.02 mm/tooth, and a cutting depth of 0.25 mm, whereas the minimum RMS V was predicted at 1000 Hz, a feed per tooth of approximately 0.032 mm/tooth, and a cutting depth of 0.50 mm. Therefore, parameter selection represents a trade-off depending on the primary technological objective. If surface quality is prioritized, the region with a lower feed per tooth and lower predicted Ra is more suitable. If vibration minimization and process stability are prioritized, conditions associated with lower RMS V are preferable. When both responses are considered simultaneously, the combined Ra + RMS V criterion represents a compromise between surface quality and vibration level. However, due to the limited predictive capability of the AlMgSi Ra model, this combined result should also be interpreted with caution and only within the investigated experimental domain.

### 4.2. Influence of Robot Configuration and Dynamic Characteristics

In robotic milling, the process is influenced not only by machining parameters but also by the dynamic characteristics of the robotic structure. The lower stiffness of industrial robots compared with conventional CNC machine tools increases their sensitivity to vibration. Makulavičius et al. [22] identified system stiffness and dynamic properties, robot arm configuration, and machining parameters among the main factors affecting the quality of robotic machining. Mousavi et al. [23] also pointed out that the dynamic properties of a robotic system vary with its configuration and the position of the manipulator within the workspace. In the present study, robot configuration was not investigated as an independent factor in the main experiment. During preliminary screening, nine positions in the XY plane were evaluated, after which position 5 was selected and maintained throughout the main experiments. The robot position should therefore be interpreted as a controlled experimental condition rather than as an independently optimized variable. Consequently, the results do not allow its influence to be quantitatively separated from the effects of the investigated machining parameters. The dynamic characteristics of the robotic assembly were evaluated before machining using experimental modal analysis. Under static conditions, without cutting and without tool-workpiece interaction, a dominant natural frequency of approximately 1.6 kHz and additional response regions at higher frequencies were identified. These results were considered when defining the spindle-speed range in relation to the two-flute tool geometry and the corresponding tooth-passing frequency, together with the operating limitations of the spindle and the results of preliminary machining experiments. However, the identified natural frequency of approximately 1.6 kHz cannot be directly interpreted as the resonance frequency of the coupled system during machining of a particular material. Once the tool engages the workpiece, the dynamic response of the system may change due to the workpiece material, workpiece and clamping stiffness, tool-workpiece interaction, and process-dependent cutting stiffness. Therefore, the actual resonance frequencies during milling were not experimentally determined in this study. The modal analysis was primarily used to support the selection of the experimental spindle-speed range.

### 4.3. Model Limitations and Future Research

Future research should focus particularly on frequency-domain analysis of vibration signals during the actual cutting process, independent experimental validation of the model-predicted conditions, and systematic variation in the robot configuration. It would also be beneficial to extend the experimental investigation to the milling of additional materials with different mechanical and technological properties, which would allow the influence of the workpiece material on the dynamic behavior of the robotic system and the quality of the machined surface to be assessed more comprehensively. Another direction for future research is the use of a more powerful high-speed spindle or a robotic manipulator with higher structural stiffness. Such modifications would allow the investigated range of machining parameters to be extended, particularly when machining metallic materials associated with higher cutting loads, while also enabling further assessment of the influence of spindle power and robotic structural stiffness on process stability. These developments could contribute to a better separation of the effects of machining parameters, material properties, and the dynamic characteristics of the robotic system, thereby improving the reliability of surface quality and process stability predictions.

## 5. Conclusions

This study investigated the influence of technological parameters on surface roughness and vibration levels during robotic milling of Corian and AlMgSi using an ABB IRB 1100 industrial robot. The combination of experimental modal analysis, full factorial design, and Response Surface Methodology (RSM) enabled the identification of favorable material-specific machining conditions.

The results demonstrated that feed per tooth, spindle rotational frequency, and cutting depth influence surface quality and process stability differently depending on the machined material. For Corian, the lowest experimentally observed surface roughness was Ra = 0.402 µm, and the developed roughness model showed good agreement with experimental data (R^2^ = 0.883). For AlMgSi, the lowest measured roughness was Ra = 0.801 µm; however, the corresponding roughness model exhibited limited predictive capability (R^2^ = 0.546). Experimental modal analysis identified a dominant natural frequency of approximately 1.6 kHz under static conditions, supporting the selection of spindle-speed ranges. The findings also confirmed that minimizing vibrations and surface roughness requires consideration of material-specific machining conditions.

The main scientific contribution of this study is the integration of dynamic characterization, experimental optimization, and statistical modeling to evaluate robotic milling performance for different materials. The obtained results provide a foundation for further investigation of materials suitable for robotic milling and for the development of predictive models and adaptive control systems. Future research will focus on integrating real-time vibration monitoring with adaptive adjustment of technological parameters, particularly spindle speed and feed rate, to improve machining stability, surface quality, and process efficiency. Further experimental validation will be required to extend these findings beyond the investigated machining conditions.

## Figures and Tables

**Table 1:** The Implementation of Industrial Robots in Various Industrial Sectors [1].

Industrial Sector	Applied Technologies	Product/Operation
Manufacturing	Milling	Rapid prototyping
Automotive	Grinding, drilling, milling, cutting, welding	Engine components, vehicle body frames, body panels, bumpers
Aerospace	Drilling, cutting, grinding, polishing	Turbine blades, propellers, insulation components, wing structures
Foundries	Milling, drilling, finishing operations	Moulds, castings
Fashion Industry	Milling, grinding	Moulds, tooling, fixtures
Shipbuilding	Milling	Ship compartments
Healthcare	Grinding, polishing	Prosthetic devices
Woodworking	Milling, material handling	Structural moulds, furniture, upholstery, railings, modelling boards

**Table 2:** Mechanical properties of Corian material for 13 mm thickness [12].

Properties	Value	Unit
Specific density	1.7	g/cm^3^
Tensile strength	34.9–39.5	N/mm^2^
Weight	23	kg/m^2^
Tensile strength upon rupture	0.36–0.49	%
Bending strength	60	N/mm^2^
Modulus of elasticity	10,000±400	N/mm^2^
Ball impact hardness	337.3±8.4	N/mm^2^

**Table 3:** Mechanical properties of EN AW-6060 T6 [12].

Properties	Value	Unit
Tensile strength	170	MPa
Yield strength Rp_0.2_	140	MPa
Elongation	6	%
Hardness HBW	70	-
Density at 20 °C	2.7	kg/dm^3^
Young’s modulus	69,500	MPa
Shear modulus	26,100	MPa

**Table 4:** Levels of technical parameters for Corian.

Factor Levels—Corian			
Levels/Technical Parameters	Rotation Frequency [Hz]	Cutting Depth [mm]	Feed per tooth [mm/tooth]
1	566.6	1.0	0.02
2	733.3	1.5	0.04
3	900.0	2.0	0.06

**Table 5:** Levels of technical parameters for AlMgSi.

Factor Levels—AlMgSi			
Levels/Technical Parameters	Rotation Frequency [Hz]	Cutting Depth [mm]	Feed per tooth [mm/tooth]
1	833.3	0.25	0.02
2	916.6	0.50	0.03
3	1000.0	-	0.04

**Table 6:** Basic characteristics of the RSM model for surface roughness (Ra) and vibration (RMS V) in machined Corian and AlMgSi materials.

Material	Response	R^2^	Adj R^2^	RMSE	n
**AlMgSi**	Ra	0.546	0.142	0.159	18
**AlMgSi**	RMS V	0.900	0.811	7.614	18
**Corian**	Ra	0.883	0.821	0.174	27
**Corian**	RMS V	0.788	0.675	10.069	27

**Table 7:** Average roughness and vibration values for Corian and AlMgSi.

Material	Criterion	Rotation Frequency [Hz]	Feed per Tooth [mm/Tooth]	Cutting Depth [mm]	Mean Ra	Mean RMS V	Score
**AlMgSi**	minimum observed Ra	1000.0	0.02	0.25	0.80	77.55	0.136
**AlMgSi**	minimum observed RMS V	1000.0	0.02	0.50	1.08	37.36	0.196
**AlMgSi**	combined observed Ra + RMS V	833.3	0.02	0.50	0.84	43.65	0.049
**Corian**	minimum observed Ra	566.7	0.02	1.50	0.40	13.34	0.007
**Corian**	minimum observed RMS V	566.7	0.02	1.00	0.55	10.93	0.045
**Corian**	combined observed Ra + RMS V	566.7	0.02	1.50	0.40	13.34	0.007

**Table 8:** RSM-predicted machining conditions for Corian and AlMgSi according to the investigated optimization criteria.

Material	Criterion	Rotation Frequency [Hz]	Feed per Tooth [mm/Tooth]	Cutting Depth [mm]	Pred Ra [µm]	Ra_CI_Low	Ra_CI_High	Pred RMSV [m/s^2^]	RMS CI Low	RMS CI High	Score
**AlMgSi**	minimum predicted Ra	833.3	0.020	0.25	0.857	0.749	0.9650	58.940	54.490	63.39	0.112
**AlMgSi**	minimum predicted RMS V	1000.0	0.032	0.50	1.244	1.112	1.3580	39.120	34.580	43.65	0.308
**AlMgSi**	combined predicted Ra + RMS V	833.3	0.020	0.25	0.857	0.749	0.9650	58.940	54.490	63.39	0.112
**Corian**	minimum predicted Ra	724.1	0.020	1.66	0.388	0.299	0.4610	26.390	23.400	29.38	0.040
**Corian**	minimum predicted RMS V	659.3	0.034	1.00	0.669	0.605	0.7410	9.114	5.557	12.67	0.094
**Corian**	combined predicted Ra + RMS V	705.6	0.020	1.33	0.403	0.309	0.4750	21.390	18.160	24.62	0.031

## Data Availability

The original contributions presented in this study are included in the article. Further inquiries can be directed to the corresponding author.
